# Practice, knowledge and attitude of physicians and pharmacists towards the spontaneous reporting system of adverse drug reactions in Switzerland

**DOI:** 10.1002/bcp.70543

**Published:** 2026-04-17

**Authors:** Fiona A. Strobel, Thomas Stammschulte, Patrick E. Beeler

**Affiliations:** ^1^ Faculty for Health Sciences and Medicine University of Lucerne Lucerne Switzerland; ^2^ Pharmacovigilance, Safety of Medicines Division Swissmedic, Swiss Agency for Therapeutic Products Berne Switzerland; ^3^ Center for Clinical Research Lucerne Cantonal Hospital/University of Lucerne Lucerne Switzerland

**Keywords:** adverse drug reactions, pharmacists, pharmacovigilance, physicians, spontaneous reporting system, Switzerland, underreporting

## Abstract

**Introduction:**

Reporting adverse drug reactions (ADRs) is essential for detecting drug risks. Despite legal obligations in Switzerland, underreporting remains an issue. This study assessed practice, knowledge and attitudes towards the spontaneous ADR reporting system among physicians and pharmacists.

**Methods:**

A nationwide cross‐sectional survey was disseminated via professional associations to physicians and pharmacists in Switzerland. The 21‐item questionnaire assessed reporting practice, knowledge, attitudes, information needs and improvement suggestions. Multivariable regression (odds ratios [ORs], 95% confidence intervals [95% CIs]) examined associations between participant characteristics and reporting habits.

**Results:**

A total of 1108 participants (834 physicians, 274 pharmacists) were included. ADRs had been suspected by 589 (53.2%), and 562 (50.7%) had reported ≥1 ADR. Most participants rejected the notion that reporting is pointless (999, 90.2%). Although 716 (64.6%) were aware of reporting obligations, 477 (43.1%) perceived reporting as time‐consuming and 270 (24.4%) reported legal concerns. About half indicated no lack of incentives (587, 53.0%) and no concerns regarding personal (580, 52.3%) or patient data protection (545, 49.2%). More than half desired clearer guidance on reportable ADRs (609, 55.0%) and 648 (58.5%) expressed interest in pharmacovigilance training. Reporting ADRs was independently associated with increasing age and training (ORs between 1.28 [95% CI: 1.15, 1.42] and 1.92 [1.32, 2.79]), whereas investing >10 min in reporting was associated with age ≥60 (OR 1.59 [1.07, 2.38]), training (OR 1.28 [1.14, 1.45]) and pharmacist status (OR 1.83 [1.34, 2.51]).

**Conclusion:**

The fundamental willingness to report ADRs despite a simultaneous lack of specific knowledge indicates a need for targeted information campaigns and training opportunities.

**What is already known about this subject**

- ADRs contribute to morbidity and mortality, and spontaneous reporting is essential for postmarketing drug safety.
- Underreporting of ADRs is common worldwide, including in Switzerland despite legal reporting obligations.

**What this study adds**

- Most physicians and pharmacists in Switzerland acknowledge the importance of ADR reporting.
- Knowledge gaps, especially regarding reportable ADRs and reporting procedures, are key barriers to reporting.
- Targeted training and clearer guidance may improve reporting.

## 1 INTRODUCTION

Adverse drug reactions (ADRs) contribute to morbidity and mortality of patients and increased healthcare system costs.^1^, ^2^, ^3^, ^4^, ^5^, ^6^ Many ADRs are only identified after the market authorization, making reliable reporting by healthcare professionals (HCPs) crucial for effective pharmacovigilance.^7^, ^8^ Reporting of ADRs allows for early detection of drug risks and the monitoring of the benefit‐harm profile of medicines.^9^

In Switzerland, HCPs are legally required to report serious or previously unknown ADRs under Article 59 of the Therapeutic Products Act (TPA).^9^, ^10^ Swissmedic, the Swiss Agency for Therapeutic Products, is responsible for drug authorization and postmarketing surveillance and coordinates the national spontaneous reporting system, including the evaluation of individual case safety reports (ICSRs) submitted by HCPs, pharmaceutical companies and patients.^11^ The reporting system is supported by regional pharmacovigilance centres, and Swissmedic is an active participant of the WHO programme for international drug monitoring.^7^ Underreporting of ADRs remains a recognized issue within spontaneous reporting systems globally that is also prevalent in Switzerland despite legal obligations.^12^, ^13^ A 2023 study revealed that over an 8‐year period, the spontaneous reporting rate in Switzerland for ADR‐related admissions was 5% and 12% for subsequent in‐hospital deaths.^14^ Factors known to contribute to ADR underreporting include insufficient training in pharmacovigilance, limited knowledge of reporting systems and attitudinal barriers among HCPs.^12^, ^15^, ^16^, ^17^ However, because of differences in healthcare systems and medical education as well as country‐specific reporting systems, the applicability of these findings to the Swiss context remains uncertain.

Addressing barriers to ADR reporting is essential to strengthen postmarket surveillance of drugs and vaccines.^16^, ^18^, ^19^ Given the substantial burden of preventable harm associated with ADRs, improving reporting practices is crucial for earlier detection and mitigation of unrecognized or insufficiently characterized risks.^7^, ^14^, ^20^

To the best of our knowledge, there are no available data on the practice, knowledge and attitudes of HCPs regarding the ADR reporting system in Switzerland. This study therefore aimed to investigate these aspects in order to inform targeted measures to improve reporting rates and the quality of ICSRs.

## 2 METHODS

The methodology of this study is reported in accordance with the Checklist for Reporting of Survey Studies (CROSS), which can be found in the appendix section 2 of this document.^21^

### 2.1 Study design

This study employed a cross‐sectional design. Cross‐sectional surveys are commonly employed to evaluate physicians' and pharmacists' practice, knowledge and attitudes regarding ADR reporting systems.^12^, ^14^, ^15^, ^16^, ^22^, ^23^

Physicians and pharmacists practicing in Switzerland constituted the target population. Participants were recruited through professional distribution channels, as no national mailing list covering all physicians or pharmacists is available in Switzerland. The survey link was disseminated via newsletters, mailing lists and internal communication channels of professional organizations, including the Swiss Medical Association (FMH), pharmaSuisse, cantonal physicians' offices and cantonal pharmacist's offices.

Our online survey was modelled after the Drug Commission of the German Medical Association's survey on side effect reporting, which was disseminated in Germany during 2017 and 2018.^24^ The 12 items of the German survey were tailored and contextualized to the local context of Switzerland. Nine additional items were designed specifically for this study, considering existing literature^12^ and setting‐specific circumstances. The questionnaire comprised a total of 21 items including both closed‐ended and open‐ended questions. Demographic information was collected from participants, and the survey instrument encompassed five key sections:

1. reporting practice,
2. knowledge of the reporting system,
3. attitude towards the reporting system,
4. information needs and
5. suggestions for improvement.

The questionnaire was translated by professional language experts, one native speaker per language, into German, French and Italian for distribution across the different language regions of the country. In the general Swiss population, the largest language group is German‐speaking (70.7%), followed by French (24.9%), Italian (4.2%) and Romansh speakers (0.2%).^25^ To ensure inclusivity, an English version was also provided. The questionnaire (available as Supporting Information) was compiled with the newest web version of Qualtrics (Qualtrics, Provo, UT, USA).

### 2.2 Pilot study

Before the survey was implemented, a pilot study to assess content validity was conducted with a group of physicians and pharmacists from the networks of the Swiss Agency for Therapeutic Products. Affiliated with pharmacovigilance departments, the pilot participants deviate from the sampled population by having above‐average awareness of ADR reporting. Experienced participants were chosen for the pilot study to validate the content, structure and relevance of the survey instrument and to prevent any oversight of critical concepts or content errors. Further, the time required to complete the questionnaire was recorded. Responses of total *n* = 13 participants (*n* = 2 physicians, *n* = 10 pharmacists, *n* = 1 with missing specified profession) informed the finalization of the questionnaire.

### 2.3 Data collection methods

The study employed different response formats tailored to each of the five key sections. (1) Reporting practice (Q1–Q3) assessed whether physicians and pharmacists had suspected or diagnosed an ADR and whether they had reported it, including the institution(s) to which the report was submitted. Response formats included categorical options (yes/no), frequency categories (never, 1–3 times, 4–10 times, >10 times) and multiple‐choice selections. (2) Knowledge of the reporting system (Q4) comprised five items addressing prior vocational training and familiarity with key aspects of ADR reporting. Items were answered using dichotomous options (yes/no). (3) Attitude towards the reporting system (Q5–Q10) included 3‐point Likert‐scale items (disagree/neutral/agree) assessing perceived barriers, a numeric free‐text item on the maximum time willing to be spent on ADR and multiple‐choice questions on preferred reporting channels, desired feedback, responses to follow‐up queries and reporting behaviour in the case of medication errors. (4) Information needs (Q11–Q13) were explored through multiple‐response questions on desired content and communication channels and a 5‐point Likert scale measuring willingness to participate in pharmacovigilance training. (5) Suggestions for improvement (Q18–Q19) were collected via open‐ended free‐text fields. To reduce non‐response, ‘no specification’ or ‘don't know’ options were provided where appropriate. Data collection involved completion of the questionnaire distributed via professional associations' contact lists, which may be considered broadly representative of the target populations. Eligibility required practice as a physician or pharmacist in Switzerland at the time of the survey. Other HCPs were not included.

### 2.4 Sample characteristics

For contextualization of participation, national workforce figures were used as reference benchmarks. At the time of the study, approximately 41 100 physicians and 8000 pharmacists were registered in Switzerland, reflecting an approximate real‐world physician‐to‐pharmacist ratio of 5:1.^26^, ^27^

### 2.5 Survey administration

Participants received an invitation for study participation and the survey link through the professional associations. Access to the survey opened on the 9th of April 2024, with data retrieval taking place on the 1st of July 2024. Before starting the survey, participants were informed about the background and aim of the project, as well as confidentiality measures and potential risks. The accompanying text further outlined the required time for participation, estimated at around 10 min. A reminder for physicians was omitted, as the distribution of the survey among physicians required more time and generated a larger response compared with pharmacists. A reminder was sent to pharmacists 2 weeks after the initial distribution. The reminder included a note that the invitation to participate can be ignored if participation has already taken place to avoid multiple participation.

### 2.6 Ethical considerations

Data collection was conducted anonymously and confidentially. A declaration of non‐responsibility from the Ethics Committee for Northwestern and Central Switzerland was obtained prior to the start of data collection (Req‐2024‐00082). Participants were informed that their participation was voluntary, that they could withdraw from the study at any time and that their data would be handled in accordance with strict data protection regulations. To preserve the anonymity of individual respondents, age group data was collected instead of a continuous number.

### 2.7 Statistical analysis

Data obtained in German, French, Italian and English were merged and analysed using R (version 4.4.1). Descriptive statistics for participant characteristics included medians and interquartile ranges for variables with a non‐normal distribution. Categorial variables are presented as frequencies and percentages.

The variable assessing the maximum number of minutes participants were willing to spend reporting an ADR was categorized into four groups (≤5, 6–10, 11–15 and >15 min) to facilitate interpretation and comparability. In addition, a threshold of >10 min was examined to distinguish respondents willing to invest more than a minimal time commitment, as 10 min represented both the sample's median and the approximate minimum time needed to complete a standard ADR report.

Comparative statistical analysis was conducted to describe differences between physicians and pharmacists: We used a Mann–Whitney *U* test to compare the distribution of continuous variables and Fisher's exact tests to compare categorical variables. Free‐text responses (Q18–Q19) were analysed using inductive thematic grouping. Responses were coded and consolidated into data‐driven categories reflecting recurring themes. The most frequently cited themes are reported to contextualize the quantitative findings. Missing data were handled using complete‐case analysis.

We used two multivariable logistic regression models to identify independent associations between participant characteristics, including vocational training, and two outcomes: (i) previous ADR reporting and (ii) willingness to invest additional time in ADR reporting. The first outcome was defined as having previously reported at least one ADR, providing insights into influencing factors that may contribute to increased reporting rates. The second outcome captured respondents' willingness to spend more than 10 min on an ADR report. Independent variables were profession, age group, sex and *Vocational training exposure*: Prior to regression modelling, pairwise associations among categorical predictors were examined using Cramér's V to assess multicollinearity. The three training‐related variables (having learned about the statutory obligation to report ADRs, about the procedure how to report and about which ADRs should be reported) showed substantial intercorrelation and therefore were combined into a single composite score (*Vocational training exposure*, range 0–3), representing the number of training domains endorsed with *Yes*. This variable was treated as numeric in both logistic regression models. Associations are presented with odds ratios (OR) and 95% confidence intervals (95% CIs). The full models are available as Supporting Information.

## 3 RESULTS

A total of 1753 participants initiated the survey. Of these, 1108 participants were included in the analysis based on their self‐reported professional background as physician or pharmacist. The final sample comprised 834 physicians (75.3%) and 274 pharmacists (24.7%). Most participants were clinically active (*n* = 1095, 98.8%), including 829 physicians working in outpatient or hospital settings and 266 pharmacists employed in public or hospital pharmacies. The analysed sample consisted of 45.8% German‐speaking, 47.2% French‐speaking and 7.0% Italian‐speaking respondents (Table 1).

**TABLE 1:** Demographics of the respondents.

	Physicians	Pharmacists	*p*	Overall
*N* (%)	834 (75.3)	274 (24.7)		1108 (100)
Gender (%)			<0.001	
Female	378 (34.1)	188 (17.0)		566 (51.1)
Male	425 (38.4)	81 (7.3)		506 (45.7)
Non‐binary/third gender	7 (0.6)	0 (0.0)		7 (0.6)
Not specified	18 (1.6)	3 (0.3)		21 (1.9)
Age (%)			<0.001	
Under 20 years	3 (0.3)	0 (0.0)		3 (0.3)
20–29 years	22 (2.0)	22 (2.0)		44 (4.0)
30–39 years	152 (13.7)	93 (8.4)		245 (22.1)
40–49 years	240 (21.7)	62 (5.6)		302 (27.3)
50–59 years	239 (21.6)	64 (5.8)		303 (27.3)
60–69 years	142 (12.8)	30 (2.7)		172 (15.5)
70 years or older	32 (2.9)	2 (0.2)		34 (3.1)
Not specified	4 (0.4)	1 (0.1)		5 (0.5)
Profession (%)				
General practitioner	275 (24.8)	0 (0.0)		275 (24.8)
Specialist in private practice	254 (22.9)	0 (0.0)		254 (22.9)
Doctor in a hospital	300 (27.1)	0 (0.0)		300 (27.1)
Other physician roles	5 (0.5)	0 (0.0)		5 (0.5)
Pharmacist in a public pharmacy	0 (0.0)	210 (19.0)		210 (19.0)
Pharmacist in a hospital pharmacy	0 (0.0)	56 (5.1)		56 (5.1)
Other pharmacist roles	0 (0.0)	8 (0.7)		8 (0.7)
Not specified	0 (0.0)	0 (0.0)		0 (0.0)
Years working since graduation (median [IQR])	20.00 [13.00, 30.00]	17.00 [9.00, 27.00]	<0.001	20.00 [11.00, 30.00]

Table 2 provides an overview of respondents' ADR reporting practice, including prior reporting experience, institutions reported to and the time effort considered acceptable for reporting.

**TABLE 2:** Reporting practice.

	Physicians	Pharmacists	*p*	Overall
*N* (%)	834 (75.3)	274 (24.7)		1108
Previously suspected or diagnosed an ADR (%)			0.001	
Yes	471 (42.5)	118 (10.6)		589 (53.2)
No	348 (31.4)	150 (13.5)		498 (44.9)
No specification/missing	15 (1.4)	6 (0.5)		21 (1.9)
Previously reported an ADR (%)			0.014	
Yes, 1–3 times	309 (27.9)	105 (9.5)		414 (37.4)
Yes, 4–10 times	68 (6.1)	17 (1.5)		85 (7.7)
Yes, more than 10 times	38 (3.4)	25 (2.3)		63 (5.7)
Never before	412 (37.2)	122 (11.0)		534 (48.2)
No specification/missing	7 (0.6)	5 (0.5)		12 (1.1)
Institutions reported to (%)				
Swissmedic	310 (28.0)	86 (7.8)	<0.001	396 (35.7)
Pharmaceutical company	87 (7.9)	45 (4.1)	0.038	132 (11.9)
Regional centre (GE, VD, TI, BS, ZH, BE)	43 (3.9)	49 (4.4)	<0.001	92 (8.3)
Cantonal doctor	15 (1.4)	1 (0.1)	0.112	16 (1.4)
Cantonal pharmacist	11 (1.0)	4 (0.4)	1.000	15 (1.4)
Hospital pharmacy/person responsible for PV	23 (2.1)	2 (0.2)	0.086	25 (2.3)
Other	34 (3.1)	7 (0.6)	0.025	41 (3.7)
Maximum number of minutes willing to spend on reporting an ADR (median [IQR])	10.00 [5.00, 15.00]	10.00 [7.00, 15.00]	<0.001	10.00 [5.00, 15.00]
≤5 min (%)	326 (29.4)	66 (6.0)		392 (35.4)
6–10 min (%)	255 (23.0)	84 (7.6)		339 (30.6)
11–15 min (%)	118 (10.6)	53 (4.8)		171 (15.4)
>15 min (%)	104 (9.4)	64 (5.8)		168 (15.2)
No specification/missing	31 (2.8)	7 (0.6)		38 (3.4)

Abbreviations: BE = Bern, BS = Basel, GE = Geneva, PV = Pharmacovigilance, TI = Ticino, VD = Vaud, ZH = Zurich.

Table 3 presents an overview of the information on ADR reporting covered during vocational training (cf. Tables ST1 and ST2 for stratified results by professional group).

**TABLE 3:** Aspects of information received as part of vocational training.

Question		Mean	Median	Most frequent answer	No. of those who responded (proportion)	Response	*n* (%)
4.1	The statutory ADR reporting obligation for healthcare professionals	0.5	Yes (1)	Yes (1)	1077 (97.2)	Yes (1)	716 (66.5)
						Do not know (0)	165 (15.3)
						No (−1)	196 (18.2)
4.2	Institutions involved in the spontaneous reporting system	0.0	Do not know (0)	Yes (1)	1053 (95.0)	Yes (1)	431 (40.9)
						Do not know (0)	239 (22.7)
						No (−1)	383 (36.4)
4.3	The procedure for reporting an adverse drug reaction	0.0	Do not know (0)	Yes (1)	1052 (94.9)	Yes (1)	438 (41.6)
						Do not know (0)	191 (18.2)
						No (−1)	423 (40.2)
4.4	Information on which adverse drug reactions should be reported	0.0	Do not know (0)	Yes (1)	1067 (96.3)	Yes (1)	448 (42.0)
						Do not know (0)	211 (19.8)
						No (−1)	408 (38.2)
4.5	Information on where I can access specialist information (e.g., swissmedicinfo.ch)	0.3	Yes (1)	Yes (1)	1063 (95.9)	Yes (1)	599 (56.3)
						Do not know (0)	151 (14.2)
						No (−1)	313 (29.4)

**FIGURE 1:**
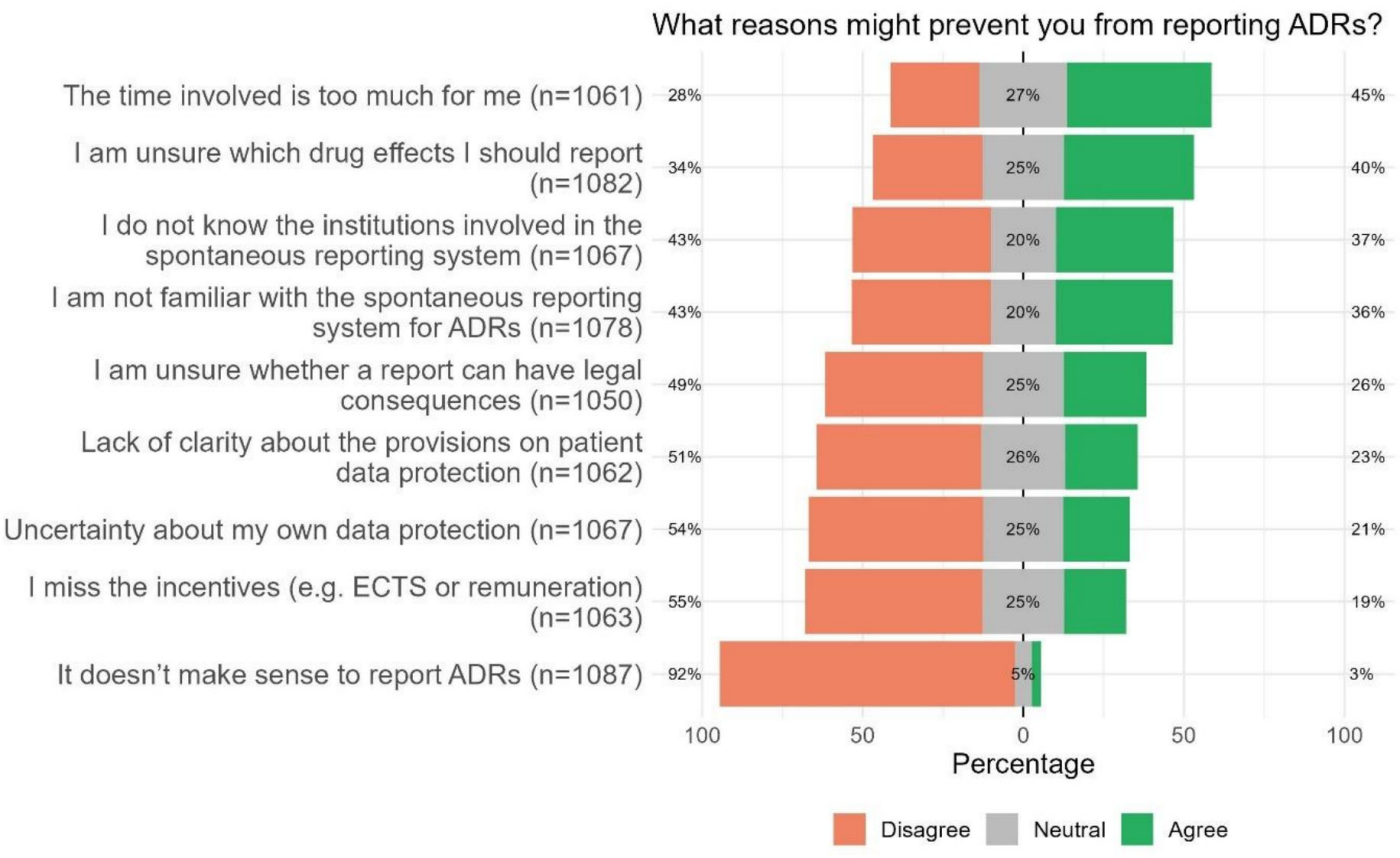
Reasons that prevent ADR reporting. ECTS = European credit transfer system. In parentheses = number of respondents/reason.

Figure 1 presents the extent to which certain issues hamper the reporting of ADRs.

Physicians reported greater unfamiliarity with the system than pharmacists (336 [41.3%] *vs*. 57 [21.5%]) and were more likely to cite this lack of familiarity as a barrier to reporting (336 [41.3%] *vs*. 57 [21.5%]). The majority of physicians and pharmacists (999 [91.9%]; 746 physicians [91.3%] and 253 [93.7%] pharmacists) disagreed with the statement that ADR reporting is meaningless. However, the perceived reporting burden varied: Nearly half of physicians (381 [47.9%]) stated the process takes too long, compared with just over a third (96 [36.1%]) of pharmacists. More than half of all participants (587 [55.2%]) did not feel that incentives for reporting were lacking, with pharmacists showing less concern in this regard (164 [61.7%]) compared with physicians (423 [53.1%]).

Detailed information on reasons that prevent ADR reporting is provided in the Supporting Information: overall (cf. Table ST3) and stratified by professional group (Tables ST4 and ST5 and Figures SF1 and SF2).

Table 4 summarizes respondents' preferences regarding ADR reporting and their related information needs.

**TABLE 4:** Preferences regarding ADR reports and information needs.

No. of responses	Percent of total	Response	Physicians	Pharmacists	Overall (% of those who responded) (multiple answers possible)
		Preferences regarding ways to report an ADR			
1107	99.9%	Online registration form via website	648 (77.7)	217 (79.2)	865 (78.1)
		Via an electronic portal	389 (46.6)	158 (57.7)	547 (49.4)
		By e‐mail	252 (30.2)	76 (27.7)	328 (29.6)
		Practice software/pharmacy software	189 (22.7)	88 (32.1)	277 (25.0)
		Via an app	139 (16.7)	55 (20.1)	194 (17.5)
		By telephone	97 (11.6)	31 (11.3)	128 (11.6)
		Registration form by post	42 (5.0)	4 (1.5)	46 (4.2)
		Registration form by fax	19 (2.3)	9 (3.3)	28 (2.5)
		Otherwise	18 (2.2)	7 (2.6)	25 (2.3)
		No specification/missing	1 (0.1)	0 (0.0)	1 (0.1)
		Measures considered helpful when reporting an ADR			
1105	99.7%	Confirmation of receipt	544 (65.2)	198 (72.3)	742 (67.1)
		Assessment of the degree of severity/causal relationship	537 (64.4)	200 (73.0)	737 (66.7)
		Information on how often this adverse drug reaction has already been reported	532 (63.8)	182 (66.4)	714 (64.6)
		Recommendations for further therapy	522 (62.6)	181 (66.1)	703 (63.6)
		Information on the suspected medicinal product (e.g., summary of product characteristics)	389 (46.6)	158 (57.7)	547 (49.5)
		Information on what happens to the reported data (processing status)	150 (18.0)	68 (24.8)	218 (19.7)
		Information on drug effects (e.g., database research and scientific publication)	136 (16.3)	55 (20.1)	191 (17.3)
		I do not need a reply	26 (3.1)	4 (1.5)	30 (2.7)
		Alternative	7 (0.8)	5 (1.8)	12 (1.1)
		No specification/missing	2 (0.2)	1 (0.4)	3 (0.3)
		Approval of statements regarding queries during the processing of an ADR report			
1098	99.1%	I would be prepared to answer specific questions in writing	488 (58.5)	188 (68.6)	676 (61.6)
		I would provide further documents on request (e.g., discharge reports and laboratory results)	508 (60.9)	141 (51.5)	649 (59.1)
		I find the demand for additional information annoying	187 (22.4)	33 (12.0)	220 (20.0)
		I do not usually answer queries	32 (3.8)	6 (2.2)	38 (3.5)
		No specification/missing	9 (1.1)	1 (0.4)	10 (0.9)
		Willingness to report ADR caused by a medication error			
1020	92.1%	Yes	503 (60.3)	173 (63.1)	676 (66.3)
		Only anonymously	186 (22.3)	35 (12.8)	221 (21.7)
		No	78 (9.4)	45 (16.4)	123 (12.1)
		No specification/missing	67 (8.0)	21 (7.7)	88 (8.6)
1104	99.6%	Information needs on the existing spontaneous reporting system			
		Information on which drug effects should be reported	481 (57.7)	128 (46.7)	609 (55.2)
		The existing options for reporting ADRs	443 (53.1)	97 (35.4)	540 (48.9)
		The procedure for reporting ADRs	444 (53.2)	96 (35.0)	540 (48.9)
		The effect of such reports	376 (45.1)	127 (46.4)	503 (45.6)
		The legal obligation of physicians and pharmacists to report	346 (41.5)	53 (19.3)	399 (36.1)
		Information on the relevance of ADRs specific to my specialty	299 (35.9)	88 (32.1)	387 (35.1)
		The legal basis for reporting ADRs	296 (35.5)	75 (27.4)	371 (33.6)
		Data privacy aspects	221 (26.5)	69 (25.2)	290 (26.3)
		The purpose of reporting ADRs	151 (18.1)	40 (14.6)	191 (17.3)
		I do not need any further information	21 (2.5)	6 (2.2)	27 (2.4)
		No specification/missing	3 (0.4)	1 (0.4)	4 (0.4)
1008	91.0%	Preferences regarding channels to receive more information about the spontaneous reporting system and its purpose			
		E‐mail newsletter	373 (44.7)	156 (56.9)	529 (52.5)
		Further training	333 (39.9)	93 (33.9)	426 (42.3)
		Monthly medical journal	310 (37.2)	31 (11.3)	341 (33.8)
		pharmaJournal	23 (2.8)	130 (47.4)	153 (15.2)
		Alternative	39 (4.7)	6 (2.2)	45 (4.5)
		I do not wish to receive any further information	21 (2.5)	6 (2.2)	27 (2.7)
		No specification/missing	73 (8.8)	27 (9.9)	100 (9.9)

*Note*: Multiple answers are possible.

The interest in further training on pharmacovigilance was strong overall (59.2%) (cf. Table ST6) and especially high among pharmacists (71.6%) (cf. Table ST8) compared with physicians (55.2%) (cf. Table ST7).

Regression showed that, compared with the youngest group of participants, the odds of having reported increased with age (Table ST9): Participants aged 40–49 had an OR of 1.52 (95% CI 1.09–2.12), those aged ≥60 an OR of 1.92 (95% CI 1.32–2.79), independent of profession. For each additional training domain in which participants reported having received instruction (statutory obligation, reporting procedure or reportable ADRs), the odds of having previously submitted an ADR report increased (OR 1.28, 95% CI 1.15–1.42).

For the outcome ‘willingness to invest more than 10 minutes’ (Table ST10), pharmacists (OR 1.83, 95% CI 1.34–2.51) were more likely than physicians to invest this amount of time. However, participants aged 50–59 years showed a trend (OR 1.39, 95% CI 0.97–2.00), participants aged 60 years and older were statistically significantly associated with investing more than 10 min in reporting an ADR (OR 1.59, 95% CI 1.07–2.38), independent of profession. For each additional training domain in which participants had received instruction, the odds of being willing to invest more than 10 min increased (OR 1.28, 95% CI 1.14–1.45).

## 4 DISCUSSION

### 4.1 Summary of the most important findings

#### 4.1.1 Reporting practice

In this nationwide analysis of the practice, knowledge and attitude of physicians and pharmacists towards the spontaneous reporting system of ADRs in Switzerland, we were able to include physicians and pharmacists with varying levels of experience in diagnosing and reporting ADRs. Although nearly all participants were clinically active, differences in specialty, clinical exposure and case mix may have influenced opportunities to detect or suspect ADRs. Regression analysis revealed that the youngest participants were associated with never having reported an ADR, whereas the odds of having reported steadily increased with age. Vocational training was statistically significantly associated with ADR reporting. This highlights the need to more explicitly integrate pharmacovigilance into Swiss medical education, which is guided by the nationally binding competency‐based PROFILES framework,^28^ defining graduate competencies and entrustable professional activities (EPAs). The ongoing revision of this framework (PROFILES 2)^29^ provides an opportunity to incorporate ADR‐related topics and reporting as core competencies. The overall median time spent on reporting was 10 min. Regression showed that pharmacists were statistically significantly associated with spending more than 10 min, independent of other characteristics. The odds of investing more than 10 min increased for the age groups 50–59 and ≥60, with the latter showing a statistically significant association with a higher time investment.

#### 4.1.2 Knowledge gaps and barriers

The results indicate a knowledge gap around how and which drug effects should be reported, particularly among physicians. Addressing these foundational training deficits could lead to improvements in engaging with the reporting system. Swissmedic has previously recognized this matter and published an explanatory video on how to correctly report ADRs for HCPs.^30^ Our findings underscore the potential to increase ADR reporting by HCPs if structural support and guidance are in place.

#### 4.1.3 Preferences and future needs

The results underscore the motivational value of response mechanisms for HCPs, with about two‐thirds of participants expressing a desire for medical‐professional feedback on their report. The need for professional feedback on ADR reports may also indicate difficulties in the diagnosis and treatment of ADRs, which are relatively rare in everyday clinical practice. This could be another important area for targeted training and contributes to an improvement in the knowledge of pharmacovigilance principles. Although most participants expressed general confidence in data protection, concerns about legal liability remain. Around one‐quarter reported fear of potential legal consequences related to ADR reporting. These findings suggest that legal concerns may still hinder full engagement with the spontaneous reporting system. Anonymous reporting could potentially help mitigate this barrier, as indicated by nearly a quarter of physicians, who would only report an ADR resulting from a medication error anonymously. Additionally, clear information on legal issues relating to ADRs and data protection aspects can help to allay existing concerns.

### 4.2 Comparison of the findings with existing literature

Consistent with previous studies,^24^ the majority of respondents acknowledged the importance of ADR reporting. Knowledge, attitudes and the extent of training were previously identified as key determinants for reporting behaviour.^15^, ^31^ These factors align with our finding that physicians, who demonstrated lower familiarity with the reporting system, had a lower reporting rate compared to pharmacists.

Our observation that the time required to complete an ADR is the most significant barrier to reporting contributes to the mixed evidence on the relevance of time constraints for pharmacists.^16^, ^32^, ^33^ However, it aligns with findings from a German study that also identified time constraints as a major barrier to reporting among physicians.^24^ Similar barriers were reported in an earlier Dutch physician survey, where uncertainty about causality, limited knowledge of reporting criteria and procedures and lack of time were among the most frequently cited reasons for non‐reporting.^34^ The time factor seems to have become increasingly important in the decision not to report cases. In an older German survey of doctors conducted in 2002, only about one‐third cited this as the reason for not reporting.^35^

Between 2009 and 2023, financial reimbursement was also more frequently cited as a reason for not reporting ADRs.^36^ In contrast, the absence of incentives did not emerge as a major barrier in this study, likely reflecting Switzerland's regulatory framework, where HCPs are legally required to report serious or unknown ADRs. Concerns about patient confidentiality, which emerged as new reasons for underreporting in a 2023 review,^36^ also did not appear to play a significant role in our context.

The reception of feedback from regulatory authorities on the submitted ICSRs has been assessed as meaningful in previous literature,^17^, ^36^ a finding confirmed by this study. Reception of personalized feedback on the submitted report has been found to increase the willingness to report,^37^ which was also stated to be helpful and relevant or, on the contrary, to be disappointing if missing in this study. In line with our findings, a survey of UK hospital pharmacists reported strong perceived professional responsibility to report ADRs and also uncertainty about reporting criteria and time constraints, whereas education and training were significant predictors of reporting participation.^38^

Participants require proper guidance on the definition of which cases should be reported, as also demonstrated in previous studies.^24^ This is consistent with Eland et al.,^34^ who found substantial uncertainty among physicians regarding which ADRs should be reported and how to report them. The identified uncertainty whether an effect with unclear attributability should be reported reinforces this conclusion. Meeting the information needs on the subject matter of physicians and pharmacists reporting obligation is of special importance, as ADRs should be reported even when in doubt about the causality.^39^ The reporting obligation according to the Swiss TPA clearly refers to unknown and serious adverse reactions. The latter criterion however may be considered too broad by HCPs to be consistently implemented in everyday clinical practice, so clearer recommendations are needed.

Although many participants reported satisfaction with the current reporting options, several strategies to enhance ADR reporting were proposed, which are in line with previous studies.^5^, ^24^, ^33^ Frequent suggestions included simplifying the reporting process and reducing reporting time, increasing information and awareness and implementing structured feedback mechanisms (cf. Table ST11). Other suggestions involved integrating tools into clinical software and strengthening the patient's role in reporting. Emerging applications of artificial intelligence (AI) hold considerable potential for automating the detection and reporting of ADRs based on administrative hospital data, as discussed by regulatory initiatives.^40^

These proposals are closely linked to the perceived time burden and excessive workload, especially among physicians, which were identified as key barriers to ADR reporting. The frequently expressed need for a more streamlined and user‐friendly reporting system reinforces findings from previous research^15^, ^36^ and underscores the importance of simplifying procedures and providing targeted support to encourage more consistent and effective engagement with ADR reporting. Our findings possibly also suggest that physicians may prefer to delegate ADR reporting or to share the workload with other professionals—such as hospital pharmacists—due to their limited ability to invest the necessary time, an approach whose benefits were emphasized by participants.

### 4.3 Implications

To increase the reporting rate, training should be focused on younger and less experienced HCPs; additional information should be practical, easily accessible and quickly understandable. Providing training on the reporting procedure and which ADRs should be reported are promising strategies to increase the number of ICSRs.^19^, ^41^, ^42^ Evidence from hospital pharmacists similarly suggests that education and training meaningfully influence engagement with spontaneous reporting systems and are important for sustaining and increasing reporting activity.^38^

To support more complete ADR reporting, physicians may benefit from structures that reduce the time burden associated with preparing ICSRs. Evidence suggests that using structured clinical data and semi‐automated approaches can substantially reduce ICSR preparation time while increasing ADR reporting.^43^ Technological improvements and increased user friendliness of the reporting system might increase the willingness of pharmacists and physicians to report. To fulfil the need for an efficient solution, closer integration of reporting options with local clinic information systems could prove effective. Mentions of missing or inappropriate input fields, restricted character input options and laborious navigation indicate that the ergonomics of the current reporting portal should be revised to make it more appealing for the user to interact with. Linkage with existing databases could streamline the process by automatically filling in information. Incorporating user‐friendly technological features could address user experience needs such as simplified menu navigation and streamlined information verification, thereby meeting the overarching demand for a simpler solution. Including a feature for anonymous reporting would further accommodate the preference to report an ADR caused by a medication error while protecting the identity of the primary notifier. Addressing general legal concerns through clear communication efforts may be necessary to support reporting behaviour.

Pharmacists overall show a greater willingness to invest time in reporting ADRs and responding to queries, as reflected in the higher reporting rates. Delegating reporting tasks to a designated pharmacovigilance contact person could help ease the administrative burden, reduce the time investment of the original reporter and increase the quality of ICSRs. Establishing a centralized entity responsible for ADR submissions on behalf of healthcare collectives may offer an effective strategy to streamline the process, ensure consistency and ultimately improve reporting outcomes.

Further research should focus on the reporting practice, pharmacovigilance knowledge and attitude of other professional groups like nurses, who are also involved in medication management.^44^ In addition, emerging approaches for the automated identification of ADRs using administrative hospital data, as well as systems that enable automatic reporting, represent promising directions for future research and should be further explored.

### 4.4 Limitations and strengths

This study has several limitations that must be considered when interpreting the findings. First, the sample was limited to physicians and pharmacists, which restricts the generalizability of the results to other professional groups. Second, because of the use of newsletter‐based and institutional distribution channels, the exact number of individuals who received or noticed the survey invitation could not be determined. Consequently, precise response rates based on invitations sent could not be calculated. If response rates were calculated using national workforce figures as reference benchmarks, they would correspond to approximately 2.0% for physicians and 3.4% for pharmacists.^26^, ^27^ However, these conservative estimates should be interpreted with caution. Not all newsletter recipients may have been aware of the survey invitation, and exposure likely varied across distribution channels. The low estimated response rates raise the possibility of non‐response bias, as physicians and pharmacists with greater interest or experience in pharmacovigilance may have been more likely to participate. In addition, reminders were sent only to pharmacists and not to physicians, which may have contributed to the higher estimated response proportion among pharmacists. However, differences may also reflect structural factors, including the substantially larger physician workforce and the fact that not all physicians are directly involved in medication dispensing or administration. Furthermore, the approximate real‐world pharmacist‐to‐physician ratio of 1:5 is not reproduced in the study sample, and the linguistic distribution differed from that of the general Swiss population, with French‐ and Italian‐speaking participants being overrepresented and German‐speaking participants underrepresented. These factors may limit the representativeness of the findings. In this study, willingness to spend more time on preparing an ADR report was included as an exploratory indicator of reporting engagement. Although greater time investment could facilitate more detailed reporting, it does not necessarily translate into higher quality ICSRs, and this assumption should therefore be interpreted with caution. Despite these limitations, the study has important strengths. The nationwide dissemination through major professional associations and official cantonal authorities suggests broad exposure within the target professions. The professional translation of the survey instrument and its adaptation to the Swiss context further strengthen the validity of the data. The findings provide valuable insights into physicians' and pharmacists' knowledge, attitudes and practices regarding the spontaneous reporting system for ADRs in Switzerland. To our knowledge, this is the first study to examine how Swiss physicians and pharmacists perceive this system and its impact on their reporting behaviour, offering relevant implications for strengthening postmarketing pharmacovigilance.

## 5 CONCLUSION

This nationwide survey revealed that physicians and pharmacists recognize the importance of reporting ADRs and are generally aware of their formal obligation to do so. However, actual reporting remains limited. Our findings indicate that this gap is attributable not only to time constraints but more importantly to persisting information deficits, with particular uncertainty about reportable ADRs and the reporting process, especially among physicians.

Regression analysis suggests that vocational training on obligation, procedures and criteria may increase reporting activity. Older professionals and pharmacists were associated with greater time investment in reporting.

Improving spontaneous ADR reporting depends on feasible reporting processes, clear guidance and targeted support for HCPs. Reducing knowledge gaps and reporting burden is likely to increase reporting participation and strengthen postmarketing drug safety surveillance.

## AUTHOR CONTRIBUTIONS

F.A.S., P.E.B. and T.S. conceived and designed the study. F.A.S. and P.E.B. processed the data and performed the statistical analyses. All authors interpreted the data. F.A.S. drafted the work with P.E.B. and T.S. critically commenting on it. All authors approved the final submitted version of the manuscript.

## CONFLICT OF INTEREST STATEMENT

F.A.S. and T.S. report no conflicts of interest. Dr. Beeler reports having received fees and funding from AstraZeneca for matters unrelated to the present study.

## CODE AVAILABILITY

The code of this study is available from the corresponding author upon reasonable request.

## Supporting information


**Table S1.** Aspects of information received as part of vocational training physicians.
**Table S2.** Aspects of information received as part of vocational training pharmacists.
**Table S3.** Reasons that prevent from ADR reporting (overall).
**Figure S1.** Reasons that prevent from ADR reporting for physicians.
**Table S4.** Reasons that prevent from ADR reporting (Physicians).
**Figure S2.** Reasons that prevent from ADR reporting for pharmacists.
**Table S5.** Reasons that prevent from ADR reporting (Pharmacists).
**Table S6.** Willingness to uptake further training in pharmacovigilance.
**Table S7.** Willingness to uptake further training in pharmacovigilance physicians.
**Table S8.** Willingness to uptake further training in pharmacovigilance pharmacists.
**Table S9.** Multivariable regression model to identify independent associations between participant characteristics incl. vocational training and having previously reported one or more ADRs.
Table S10. Multivariable regression model to identify independent associations between participant characteristics incl. vocational training and willingness to spend more time on reporting an ADR.
**Table S11.** List of feedback from the free‐text responses to the survey regarding.

## Data Availability

Supplementary tables are available with this publication. The data that support the findings of this study are available on reasonable request from the corresponding author.
