# Supplementary material for: Practice, knowledge and attitude of physicians and pharmacists towards the spontaneous reporting system of adverse drug reactions in Switzerland
Source: Br J Clin Pharmacol. 2026 Apr 17;92(8):2797–808. doi: 10.1002/bcp.70543 (PMC13421059; doi:10.1002/bcp.70543)
Supplement: Supplementary file 1 — Table S1. Aspects of information received as part of vocational training physicians. Table S2. Aspects of information received as part of vocational training pharmacists. Table S3. Reasons that prevent from ADR reporting (overall). Figure S1. Reasons that prevent from ADR reporting for physicians. Table S4. Reasons that prevent from ADR reporting (Physicians). Figure S2. Reasons that prevent from ADR reporting for pharmacists. Table S5. Reasons that prevent from ADR reporting (Pharmacists). Table S6. Willingness to uptake further training in pharmacovigilance. Table S7. Willingness to uptake further training in pharmacovigilance physicians. Table S8. Willingness to uptake further training in pharmacovigilance pharmacists. Table S9. Multivariable regression model to identify independent associations between participant characteristics incl. vocational training and having previously reported one or more ADRs. Table S10. Multivariable regression model to identify independent associations between participant characteristics incl. vocational training and willingness to spend more time on reporting an ADR. Table S11. List of feedback from the free‐text responses to the survey regarding. [file BCP-92-2797-s001.docx]

Supplementary Table ST1. Aspects of information received as part of vocational training physicians

| **Question** |  | **Mean** | **Median** | **Most frequent answer** | **N responded (Proportion)** | **Response** | **n (%)** |
| --- | --- | --- | --- | --- | --- | --- | --- |
| 4.1 | The statutory ADR reporting obligation for healthcare professionals | 0.4 | Yes (1) | Yes (1) | 808 (96.9) | Yes (1) | 483 (59.8) |
|  |  |  |  |  |  | Don't know (0) | 150 (18.6) |
|  |  |  |  |  |  | No (-1) | 175 (21.7) |
| 4.2 | Institutions involved in the spontaneous reporting system | -0.1 | Don't know (0) | No (-1) | 789 (94.6) | Yes (1) | 274 (34.7) |
|  |  |  |  |  |  | Don't know (0) | 193 (24.5) |
|  |  |  |  |  |  | No (-1) | 322 (40.8) |
| 4.3 | The procedure for reporting an adverse drug reaction | -0.1 | Don't know (0) | No (-1) | 787 (94.4) | Yes (1) | 271 (34.4) |
|  |  |  |  |  |  | Don't know (0) | 161 (20.5) |
|  |  |  |  |  |  | No (-1) | 355 (45.1) |
| 4.4 | Information on which adverse drug reactions should be reported | -0.1 | Don't know (0) | No (-1) | 799 (95.8) | Yes (1) | 289 (36.2) |
|  |  |  |  |  |  | Don't know (0) | 173 (21.7) |
|  |  |  |  |  |  | No (-1) | 337 (42.2) |
| 4.5 | Information on where I can access specialist information (e.g. [swissmedicinfo.ch](http://swissmedicinfo.ch)) | 0.2 | Yes (1) | Yes (1) | 795 (95.3) | Yes (1) | 405 (50.9) |
|  |  |  |  |  |  | Don't know (0) | 130 (16.4) |
|  |  |  |  |  |  | No (-1) | 260 (32.7) |

Supplementary Table ST2. Aspects of information received as part of vocational training pharmacists

| **Question** |  | **Mean** | **Median** | **Most frequent answer** | **N responded (Proportion)** | **Response** | **n (%)** |
| --- | --- | --- | --- | --- | --- | --- | --- |
| 4.1 | The statutory ADR reporting obligation for healthcare professionals | 0.8 | Yes (1) | Yes (1) | 269 (98.2) | Yes (1) | 233 (86.6) |
|  |  |  |  |  |  | Don't know (0) | 15 (5.6) |
|  |  |  |  |  |  | No (-1) | 21 (7.8) |
| 4.2 | Institutions involved in the spontaneous reporting system | 0.4 | Yes (1) | Yes (1) | 264 (96.4) | Yes (1) | 157 (59.5) |
|  |  |  |  |  |  | Don't know (0) | 46 (17.4) |
|  |  |  |  |  |  | No (-1) | 61 (23.1) |
| 4.3 | The procedure for reporting an adverse drug reaction | 0.4 | Yes (1) | Yes (1) | 265 (96.7) | Yes (1) | 167 (63.0) |
|  |  |  |  |  |  | Don't know (0) | 30 (11.3) |
|  |  |  |  |  |  | No (-1) | 68 (25.7) |
| 4.4 | Information on which adverse drug reactions should be reported | 0.3 | Yes (1) | Yes (1) | 268 (97.8) | Yes (1) | 159 (59.3) |
|  |  |  |  |  |  | Don't know (0) | 38 (14.2) |
|  |  |  |  |  |  | No (-1) | 71 (26.5) |
| 4.5 | Information on where I can access specialist information (e.g. [swissmedicinfo.ch](http://swissmedicinfo.ch)) | 0.5 | Yes (1) | Yes (1) | 268 (97.8) | Yes (1) | 194 (72.4) |
|  |  |  |  |  |  | Don't know (0) | 21 (7.8) |
|  |  |  |  |  |  | No (-1) | 53 (19.8) |

Supplementary Table ST3. Reasons that prevent from ADR reporting (overall)

| **Question** |  | **Mean** | **Median** | **Most frequent answer** | **N responded (Proportion)** | **Response** | **n (%)** |
| --- | --- | --- | --- | --- | --- | --- | --- |
| 5.1 | The time involved is too much for me. | 0.2 | Neutral (0) | Agree (1) | 1061 (95.8) | Agree (1) | 477 (45.0) |
|  |  |  |  |  |  | Neutral (0) | 290 (27.3) |
|  |  |  |  |  |  | Disagree (-1) | 294 (27.7) |
| 5.2 | I am not familiar with the spontaneous reporting system for adverse drug reactions. | -0.1 | Neutral (0) | Disagree (-1) | 1078 (97.3) | Agree (1) | 393 (36.5) |
|  |  |  |  |  |  | Neutral (0) | 218 (20.2) |
|  |  |  |  |  |  | Disagree (-1) | 393 (36.5) |
| 5.3 | I do not know the institutions involved in the spontaneous reporting system. | -0.1 | Neutral (0) | Disagree (-1) | 1067 (96.3) | Agree (1) | 391 (36.6) |
|  |  |  |  |  |  | Neutral (0) | 217 (20.3) |
|  |  |  |  |  |  | Disagree (-1) | 459 (43.0) |
| 5.4 | I don't think it makes sense to report ADRs. | -0.9 | Disagree (-1) | Disagree (-1) | 1087 (98.1) | Agree (1) | 31 (2.9) |
|  |  |  |  |  |  | Neutral (0) | 57 (5.2) |
|  |  |  |  |  |  | Disagree (-1) | 999 (91.9) |
| 5.5 | I miss the incentives (e.g. ECTS or remuneration). | -0.4 | Disagree (-1) | Disagree (-1) | 1063 (95.9) | Agree (1) | 205 (19.3) |
|  |  |  |  |  |  | Neutral (0) | 271 (25.5) |
|  |  |  |  |  |  | Disagree (-1) | 587 (55.2) |
| 5.6 | I am unsure which drug effects I should report. | 0.1 | Neutral (0) | Agree (1) | 1082 (97.7) | Agree (1) | 438 (40.5) |
|  |  |  |  |  |  | Neutral (0) | 274 (25.3) |
|  |  |  |  |  |  | Disagree (-1) | 370 (34.2) |
| 5.7 | Lack of clarity about the provisions on patient data protection | -0.3 | Disagree (-1) | Disagree (-1) | 1062 (95.8) | Agree (1) | 239 (22.5) |
|  |  |  |  |  |  | Neutral (0) | 278 (26.2) |
|  |  |  |  |  |  | Disagree (-1) | 545 (51.3) |
| 5.8 | Uncertainty about my own data protection | -0.3 | Disagree (-1) | Disagree (-1) | 1067 (96.3) | Agree (1) | 221 (20.7) |
|  |  |  |  |  |  | Neutral (0) | 266 (24.9) |
|  |  |  |  |  |  | Disagree (-1) | 580 (54.4) |
| 5.9 | I am unsure whether a report can have legal consequences. | -0.2 | Neutral (0) | Disagree (-1) | 1050 (94.8) | Agree (1) | 270 (25.7) |
|  |  |  |  |  |  | Neutral (0) | 264 (25.1) |
|  |  |  |  |  |  | Disagree (-1) | 516 (49.1) |

Supplementary Figure SF1. Reasons that prevent from ADR reporting for physicians


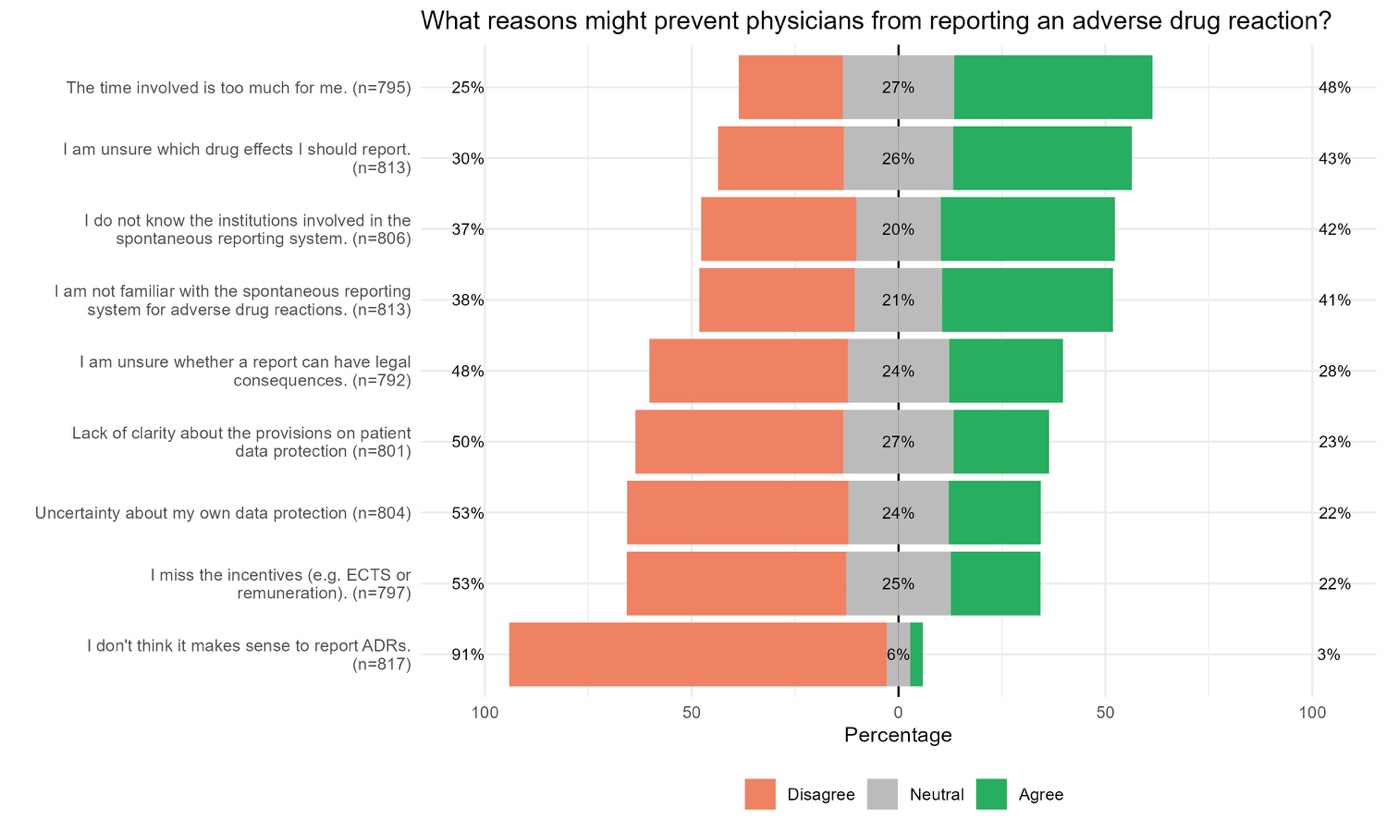


Supplementary Figure 1: Reasons that prevent from ADR reporting for **Physicians**

Supplementary Table ST4. Reasons that prevent from ADR reporting (Physicians)

| **Question** |  | **Mean** | **Median** | **Most frequent answer** | **N responded (Proportion)** | **Response** | **n (%)** |
| --- | --- | --- | --- | --- | --- | --- | --- |
| 5.1 | The time involved is too much for me. | 0.2 | Neutral (0) | Agree (1) | 794 (95.2) | Agree (1) | 380 (47.9) |
|  |  |  |  |  |  | Neutral (0) | 241 (27.0) |
|  |  |  |  |  |  | Disagree (-1) | 200 (25.2) |
| 5.2 | I am not familiar with the spontaneous reporting system for adverse drug reactions. | 0.0 | Neutral (0) | Agree (1) | 812 (97.4) | Agree (1) | 336 (41.4) |
|  |  |  |  |  |  | Neutral (0) | 170 (20.9) |
|  |  |  |  |  |  | Disagree (-1) | 306 (37.6) |
| 5.3 | I do not know the institutions involved in the spontaneous reporting system. | 0.0 | Neutral (0) | Agree (1) | 805 (96.5) | Agree (1) | 339 (42.1) |
|  |  |  |  |  |  | Neutral (0) | 164 (20.4) |
|  |  |  |  |  |  | Disagree (-1) | 302 (37.5) |
| 5.4 | I don't think it makes sense to report ADRs. | -0.9 | Disagree (-1) | Disagree (-1) | 816 (97.8) | Agree (1) | 25 (3.1) |
|  |  |  |  |  |  | Neutral (0) | 45 (5.5) |
|  |  |  |  |  |  | Disagree (-1) | 746 (91.4) |
| 5.5 | I miss the incentives (e.g. ECTS or remuneration). | -0.3 | Disagree (-1) | Disagree (-1) | 796 (95.4) | Agree (1) | 173 (21.7) |
|  |  |  |  |  |  | Neutral (0) | 200 (25.1) |
|  |  |  |  |  |  | Disagree (-1) | 423 (53.1) |
| 5.6 | I am unsure which drug effects I should report. | 0.1 | Neutral (0) | Agree (1) | 812 (97.4) | Agree (1) | 351 (43.2) |
|  |  |  |  |  |  | Neutral (0) | 214 (26.4) |
|  |  |  |  |  |  | Disagree (-1) | 247 (30.4) |
| 5.7 | Lack of clarity about the provisions on patient data protection | -0.3 | Disagree (-1) | Disagree (-1) | 800 (95.9) | Agree (1) | 185 (23.1) |
|  |  |  |  |  |  | Neutral (0) | 212 (26.5) |
|  |  |  |  |  |  | Disagree (-1) | 403 (50.4) |
| 5.8 | Uncertainty about my own data protection | -0.3 | Disagree (-1) | Disagree (-1) | 803 (96.3) | Agree (1) | 179 (22.3) |
|  |  |  |  |  |  | Neutral (0) | 194 (24.2) |
|  |  |  |  |  |  | Disagree (-1) | 430 (53.5) |
| 5.9 | I am unsure whether a report can have legal consequences. | -0.2 | Neutral (0) | Disagree (-1) | 791 (94.8) | Agree (1) | 218 (27.6) |
|  |  |  |  |  |  | Neutral (0) | 193 (24.4) |
|  |  |  |  |  |  | Disagree (-1) | 380 (48.0) |

Supplementary Figure SF2. Reasons that prevent from ADR reporting for pharmacists


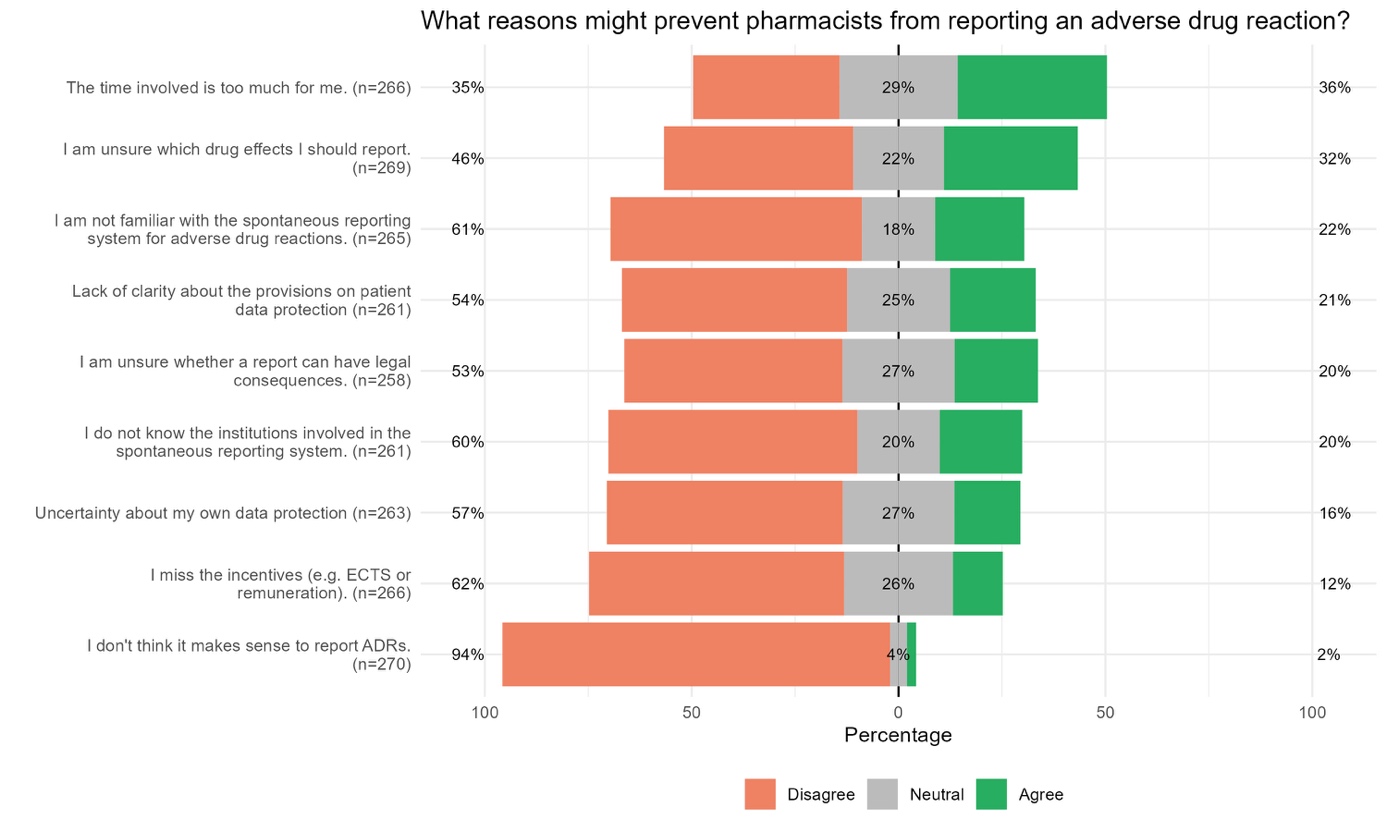


Supplementary Figure 2: Reasons that prevent from ADR reporting for **Pharmacists**

Supplementary Table ST5. Reasons that prevent from ADR reporting (Pharmacists)

| **Question** |  | **Mean** | **Median** | **Most frequent answer** | **N responded (Proportion)** | **Response** | **n (%)** |
| --- | --- | --- | --- | --- | --- | --- | --- |
| 5.1 | The time involved is too much for me. | 0.0 | Neutral (0) | Agree (1) | 267 (97.4) | Agree (1) | 97  (36.3) |
|  |  |  |  |  |  | Neutral (0) | 76 (28.5) |
|  |  |  |  |  |  | Disagree (-1) | 94 (35.2) |
| 5.2 | I am not familiar with the spontaneous reporting system for adverse drug reactions. | -0.4 | Disagree (-1) | Disagree (-1) | 266 (97.1) | Agree (1) | 57 (21.4) |
|  |  |  |  |  |  | Neutral (0) | 48 (18.0) |
|  |  |  |  |  |  | Disagree (-1) | 161 (60.5) |
| 5.3 | I do not know the institutions involved in the spontaneous reporting system. | -0.4 | Disagree (-1) | Disagree (-1) | 262 (95.6) | Agree (1) | 52 (19.8) |
|  |  |  |  |  |  | Neutral (0) | 53 (20.2) |
|  |  |  |  |  |  | Disagree (-1) | 157 59.9) |
| 5.4 | I don't think it makes sense to report ADRs. | -0.9 | Disagree (-1) | Disagree (-1) | 271 (98.9) | Agree (1) | 6 (2.2) |
|  |  |  |  |  |  | Neutral (0) | 12 (4.4) |
|  |  |  |  |  |  | Disagree (-1) | 253 (93.4) |
| 5.5 | I miss the incentives (e.g. ECTS or remuneration). | -0.5 | Disagree (-1) | Disagree (-1) | 267 (97.4) | Agree (1) | 32 (12.0) |
|  |  |  |  |  |  | Neutral (0) | 71 (26.6) |
|  |  |  |  |  |  | Disagree (-1) | 164 (61.4) |
| 5.6 | I am unsure which drug effects I should report. | -0.1 | Neutral (0) | Disagree (-1) | 270 (98.5) | Agree (1) | 87 (32.2) |
|  |  |  |  |  |  | Neutral (0) | 60 (22.2) |
|  |  |  |  |  |  | Disagree (-1) | 123 (45.6) |
| 5.7 | Lack of clarity about the provisions on patient data protection | -0.3 | Disagree (-1) | Disagree (-1) | 262 (95.6) | Agree (1) | 54 (20.6) |
|  |  |  |  |  |  | Neutral (0) | 66 (25.2) |
|  |  |  |  |  |  | Disagree (-1) | 142 (54.2) |
| 5.8 | Uncertainty about my own data protection | -0.4 | Disagree (-1) | Disagree (-1) | 264 (96.4) | Agree (1) | 42 (15.9) |
|  |  |  |  |  |  | Neutral (0) | 72 (27.3) |
|  |  |  |  |  |  | Disagree (-1) | 150 (56.8) |
| 5.9 | I am unsure whether a report can have legal consequences. | -0.3 | Disagree (-1) | Disagree (-1) | 259 (94.5) | Agree (1) | 52 (20.1) |
|  |  |  |  |  |  | Neutral (0) | 71 (27.4) |
|  |  |  |  |  |  | Disagree (-1) | 136 (52.5) |

Supplementary Table ST6. Willingness to uptake further training in pharmacovigilance

| **Profession** | **Mean** | **Median** | **Statistical peak** | **N responses** | **Percent of total** | **Response** | **N responses** | **Proportion of who responded (%)** |
| --- | --- | --- | --- | --- | --- | --- | --- | --- |
| Both | 2.6 | Rather yes (2) | Rather yes (2) | 1094 | 99.7% | Yes (1) | 210 | 19.2 |
|  |  |  |  |  |  | Rather yes (2) | 438 | 40.0 |
|  |  |  |  |  |  | Neutral (3) | 134 | 12.2 |
|  |  |  |  |  |  | Rather no (4) | 191 | 17.5 |
|  |  |  |  |  |  | No (5) | 121 | 11.1 |

Supplementary Table ST7. Willingness to uptake further training in pharmacovigilance physicians

| **Profession** | **Mean** | **Median** | **Statistical peak** | **N responses** | **Percent of professional group** | **Response** | **N responses** | **Proportion of who responded (%)** |
| --- | --- | --- | --- | --- | --- | --- | --- | --- |
| Physicians | 2.8 | Rather yes (2) | Rather yes (2) | 822 | 98.6% | Yes (1) | 122 | 14.6 |
|  |  |  |  |  |  | Rather yes (2) | 331 | 39.7 |
|  |  |  |  |  |  | Neutral (3) | 95 | 11.4 |
|  |  |  |  |  |  | Rather no (4) | 167 | 20.0 |
|  |  |  |  |  |  | No (5) | 107 | 12.8 |

Supplementary Table ST8. Willingness to uptake further training in pharmacovigilance pharmacists

| **Profession** | **Mean** | **Median** | **Statistical peak** | **N responses** | **Percent of professional group** | **Response** | **N responses** | **Proportion of who responded (%)** |
| --- | --- | --- | --- | --- | --- | --- | --- | --- |
| Pharmacists | 2.2 | Rather yes (2) | Rather yes (2) | 272 | 99.3% | Yes (1) | 88 | 32.1 |
|  |  |  |  |  |  | Rather yes (2) | 107 | 39.1 |
|  |  |  |  |  |  | Neutral (3) | 39 | 14.2 |
|  |  |  |  |  |  | Rather no (4) | 24 | 8.8 |
|  |  |  |  |  |  | No (5) | 14 | 5.1 |

Supplementary Table ST9. Multivariable regression model to identify independent associations between participant characteristics incl. vocational training and having previously reported one or more ADRs.

**Outcome: Has previously reported at least one ADR**

| **Variable** | **OR** | **(95% CI)** | **Stat. sign.** |
| --- | --- | --- | --- |
| Physicians | Ref |  |  |
| Pharmacists | 1.15 | (0.85,1.57) |  |
| 39 years old and younger | Ref |  |  |
| 40-49 years old | 1.52 | (1.09,2.12) | * |
| 50-59 years old | 1.78 | (1.28,2.49) | * |
| 60 and older | 1.92 | (1.32,2.79) | * |
| Female | Ref |  |  |
| Not female | 1.22 | (0.95,1.58) |  |
| Vocational training exposure | 1.28 | (1.15,1.42) | * |

Ref: Reference=1

OR: Odds ratio

95% CI: 95% confidence interval

Stat. sign.: Statistically significant

**Note on the composite *Vocational training* variable:**

Because the three training variables (statutory reporting obligation, reporting procedure, and reportable ADRs) were strongly correlated, they were aggregated into a single composite measure (*Vocational training exposure*). This variable counts how many of the three domains respondents answered with *Yes*, resulting in a score from 0 (no training in any domain) to 3 (training in all domains). In the regression models, this score is treated as a numeric predictor: each one-unit increase represents exposure to one additional pharmacovigilance training domain. The corresponding odds ratio therefore quantifies how the odds of the outcome change for every additional training component learned.

Supplementary Table ST10. Multivariable regression model to identify independent associations between participant characteristics incl. vocational training and willingness to spend more time on reporting an ADR.

**Outcome: Is willing to spend more than ten minutes on reporting an ADR**

| **Variable** | **OR** | **(95% CI)** | **Stat. sign.** |
| --- | --- | --- | --- |
| Physicians | Ref |  |  |
| Pharmacists | 1.83 | (1.34,2.51) | * |
| 39 years old and younger | Ref |  |  |
| 40-49 years old | 0.97 | (0.66,1.41) |  |
| 50-59 years old | 1.39 | (0.97,2.00) |  |
| 60 and older | 1.59 | (1.07,2.38) | * |
| Female | Ref |  |  |
| Not female | 0.97 | (0.73,1.28) |  |
| Vocational training exposure | 1.28 | (1.14,1.45) | * |

Ref: Reference=1

OR: Odds ratio

95% CI: 95% confidence interval

Stat. sign.: Statistically significant

**Note on the composite *Vocational training* variable:**

Because the three training variables (statutory reporting obligation, reporting procedure, and reportable ADRs) were strongly correlated, they were aggregated into a single composite measure (*Vocational training exposure*). This variable counts how many of the three domains respondents answered with *Yes*, resulting in a score from 0 (no training in any domain) to 3 (training in all domains). In the regression models, this score is treated as a numeric predictor: each one-unit increase represents exposure to one additional pharmacovigilance training domain. The corresponding odds ratio therefore quantifies how the odds of the outcome change for every additional training component learned.

Supplementary Table ST11. List of feedback from the free-text responses to the survey regarding

| **Recommendations** | **N** |
| --- | --- |
| No improvements needed | 107 |
| Simplification of access, usage, and administrative processes | 105 |
| Reduction of reporting time | 48 |
| Improved training, education, communication, and awareness of reporting relevance | 46 |
| Proposals for alternative reporting solutions or responsibility changes | 35 |
| Functional (frontend and User Interface) improvements (Elvis functional changes) | 24 |
| Enhanced feedback, causality assessment, and expert evaluation | 22 |
| System stability and performance improvements (Elvis-related issues) | 18 |
| Integration into local systems and use of innovative tools | 17 |
| Other or not relevant suggestions | 16 |
| Introduction of Incentives | 14 |
| Clarification of the importance and consequences of reporting ADRs | 10 |
| Clarification of reporting criteria and definition of ADRs | 10 |
| Establishment of a dedicated pharmacovigilance contact person or entity | 9 |
| Improved visibility of the reporting system and process information | 9 |
| Increased transparency and public access to reporting data | 7 |
| Strengthening of legal protection for reporters | 5 |
| Elvis system is satisfactory as it is | 4 |
| Stronger involvement of patients in reporting processes | 3 |
| Request to eliminate follow-up questions and feedback requests | 2 |

Checklist for Reporting Of Survey Studies (CROSS)

| **Section/topic** | **Item** | **Item description** | **Reported on page #** |
| --- | --- | --- | --- |
| **Title and abstract** | | |  |
| Title and abstract | 1a | State the word “survey” along with a commonly used term in title or abstract to introduce the study’s design. | **1** |
|  | 1b | Provide an informative summary in the abstract, covering background, objectives, methods, findings/results, interpretation/discussion, and conclusions. | **1** |
| **Introduction** | | |  |
| Background | 2 | Provide a background about the rationale of study, what has been previously done, and why this survey is needed. | **3** |
| Purpose/aim | 3 | Identify specific purposes, aims, goals, or objectives of the study. | **4** |
| **Methods** | | |  |
| Study design | 4 | Specify the study design in the methods section with a commonly used term (e.g., cross-sectional or longitudinal). | **5** |
|  | 5a | Describe the questionnaire (e.g., number of sections, number of questions, number and names of instruments used). | **6** |
| Data collection methods | 5b | Describe all questionnaire instruments that were used in the survey to measure particular concepts. Report target population, reported validity and reliability information, scoring/classification procedure, and reference links (if any). | **6** |
|  | 5c | Provide information on pretesting of the questionnaire, if performed (in the article or in an online supplement). Report the method of pretesting, number of times questionnaire was pre-tested, number and demographics of participants used for pretesting, and the level of similarity of demographics between pre-testing participants and sample population. | **6** |
|  | 5d | Questionnaire if possible, should be fully provided (in the article, or as appendices or as an online supplement). | **XVIII-XXXVII** |
| Sample characteristics | 6a | Describe the study population (i.e., background, locations, eligibility criteria for participant inclusion in survey, exclusion criteria). | **5,7** |
|  | 6b | Describe the sampling techniques used (e.g., single stage or multistage sampling, simple random sampling, stratified sampling, cluster sampling, convenience sampling). Specify the locations of sample participants whenever clustered sampling was applied. | **5** |
|  | 6c | Provide information on sample size, along with details of sample size calculation. | **12** |
|  | 6d | Describe how representative the sample is of the study population (or target population if possible), particularly for population-based surveys. | **7** |
| Survey  administration | 7a | Provide information on modes of questionnaire administration, including the type and number of contacts, the location where the survey was conducted (e.g., outpatient room or by use of online tools, such as SurveyMonkey). | **5,6** |
|  | 7b | Provide information of survey’s time frame, such as periods of recruitment, exposure, and follow-up days. | **8** |
|  | 7c | Provide information on the entry process:  –>For non-web-based surveys, provide approaches to minimize human error in data entry.  –>For web-based surveys, provide approaches to prevent “multiple participation” of participants. | **8** |
| Study preparation | 8 | Describe any preparation process before conducting the survey (e.g., interviewers’ training process, advertising the survey). | **8** |
| Ethical considerations | 9a | Provide information on ethical approval for the survey if obtained, including informed consent, institutional review board [IRB] approval, Helsinki declaration, and good clinical practice [GCP] declaration (as appropriate). | **8** |
|  | 9b | Provide information about survey anonymity and confidentiality and describe what mechanisms were used to protect unauthorized access. | **8,9** |
| Statistical  analysis | 10a | Describe statistical methods and analytical approach. Report the statistical software that was used for data analysis. | **10,11** |
|  | 10b | Report any modification of variables used in the analysis, along with reference (if available). | **10** |
|  | 10c | Report details about how missing data was handled. Include rate of missing items, missing data mechanism (i.e., missing completely at random [MCAR], missing at random [MAR] or missing not at random [MNAR]) and methods used to deal with missing data (e.g., multiple imputation). | **10** |
|  | 10d | State how non-response error was addressed. | **7** |
|  | 10e | For longitudinal surveys, state how loss to follow-up was addressed. | **NA** |
|  | 10f | Indicate whether any methods such as weighting of items or propensity scores have been used to adjust for non-representativeness of the sample. | **NA** |
|  | 10g | Describe any sensitivity analysis conducted. | **NA** |
| **Results** | | |  |
| Respondent characteristics | 11a | Report numbers of individuals at each stage of the study. Consider using a flow diagram, if possible. | **NA** |
|  | 11b | Provide reasons for non-participation at each stage, if possible. | **NA** |
|  | 11c | Report response rate, present the definition of response rate or the formula used to calculate response rate. | **5,12** |
|  | 11d | Provide information to define how unique visitors are determined. Report number of unique visitors along with relevant proportions (e.g., view proportion, participation proportion, completion proportion). | **12** |
| Descriptive  results | 12 | Provide characteristics of study participants, as well as information on potential confounders and assessed outcomes. | **12,13** |
| Main findings | 13a | Give unadjusted estimates and, if applicable, confounder-adjusted estimates along with 95% confidence intervals and p-values. | **NA** |
|  | 13b | For multivariable analysis, provide information on the model building process, model fit statistics, and model assumptions (as appropriate). | **10,11** |
|  | 13c | Provide details about any sensitivity analysis performed. If there are considerable amount of missing data, report sensitivity analyses comparing the results of complete cases with that of the imputed dataset (if possible). | **NA** |
| **Discussion** | | |  |
| Limitations | 14 | Discuss the limitations of the study, considering sources of potential biases and imprecisions, such as non-representativeness of sample, study design, important uncontrolled confounders. | **27** |
| Interpretations | 15 | Give a cautious overall interpretation of results, based on potential biases and imprecisions and suggest areas for future research. | **20,21,25,26** |
| Generalizability | 16 | Discuss the external validity of the results. |  |
| **Other sections** | | |  |
| Role of funding source | 17 | State whether any funding organization has had any roles in the survey’s design, implementation, and analysis. | **29** |
| Conflict of interest | 18 | Declare any potential conflict of interest. | **29** |
| Acknowledgements | 19 | Provide names of organizations/persons that are acknowledged along with their contribution to the research. | **29** |

## Data collection instrument in German, Italian, French, and English

***Start***

Vielen Dank für Ihre Teilnahme an der Befragung zum Thema
„**Praxis, Wissen und Einstellung von Ärzt:innen und Apotheker:innen zum Spontanmeldesystem unerwünschter Arzneimittelwirkungen in der Schweiz**“.
Vi ringraziamo per aver partecipato al sondaggio sul tema

"**Pratica, conoscenza e atteggiamento di medici e farmacisti nei confronti del sistema di segnalazione spontanea delle reazioni avverse ai farmaci in Svizzera**".

Nous vous remercions de votre participation à l'enquête sur le thème
"**Pratique, connaissances et attitude des médecins et des pharmaciens à l'égard du système d'annonce spontanée des effets indésirables des médicaments en Suisse**".

Thank you for your participation in the survey on the topic
"**Practice, knowledge and attitudes of doctors and pharmacists towards the spontaneous reporting system for adverse drug reactions in Switzerland**".

Die Befragung dauert ungefähr 10 Minuten. 
Il colloquio dura circa 10 minuti.

L'entretien dure environ 10 minutes.

Filling out the questionnaire takes about 10 minutes.

Im Folgenden werden einige Informationen zum Hintergrund des Projekts erläutert.

Di seguito vengono fornite alcune informazioni di base sul progetto.

Vous trouverez ci-dessous quelques informations sur le contexte du projet.

The following provides some background information on the project.

**Hintergrund und Ziel des Projekts**:
Die unzureichende Erfassung schwerwiegender unerwünschter Arzneimittelwirkungen (UAW) ist ein anerkanntes Problem.
Der Zweck der geplanten Erhebung besteht darin, das Wissen und die Einstellung von Ärzt:innen und Apotheker:innen in Bezug auf das Meldesystem für unerwünschte Arzneimittelwirkungen in der Schweiz zu untersuchen.

**Contesto e scopo del progetto**:
La registrazione inadeguata delle reazioni avverse gravi ai farmaci (ADR) è un problema riconosciuto. Lo scopo di questo studio è indagare la pratica, le conoscenze e gli atteggiamenti di medici e farmacisti riguardo al sistema di segnalazione delle ADR in Svizzera.

**Contexte et objectif du projet**:
L'enregistrement insuffisant des effets indésirables graves des médicaments (EI) est un problème reconnu. L'objectif de l'enquête prévue est d'examiner les connaissances et les attitudes des médecins et des pharmaciens en ce qui concerne le système d'annonce des effets indésirables des médicaments en Suisse.

**Background and aim of the project:**
The inadequate recording of serious adverse drug reactions (ADRs) is a recognized problem. The aim of this study is to investigate the practice, knowledge and attitudes of physicians and pharmacists with regard to the ADR reporting system in Switzerland.

**Nutzen: Mit dieser Befragung werden Anhaltspunkte für effiziente und evidenzbasierte Massnahmen zur Verbesserung des UAW-Meldesystems in der Schweiz identifiziert. Dieses soll so benutzerfreundlich wie möglich gestaltet werden, um den Auftrag der gesetzlichen Meldepflicht zu erfüllen.**

**Vantaggi: L'indagine consentirà di individuare i punti di riferimento per misure efficaci e basate sull'evidenza per migliorare il sistema di segnalazione delle ADR in Svizzera. Tale sistema dovrebbe essere concepito in modo da essere il più semplice possibile per soddisfare l'obbligo legale di segnalazione.**

**Utilité**: Cette enquête permet d'identifier des pistes pour des mesures efficaces et fondées sur des preuves afin d'améliorer le système de déclaration des EI en Suisse. Celui-ci doit être conçu de manière aussi conviviale que possible afin de remplir le mandat de l'obligation légale de déclaration.
**Benefit**: This survey will identify points of reference for efficient and evidence-based measures to improve the ADR reporting system in Switzerland. This should be designed to be as user-friendly as possible in order to fulfill the legal reporting obligation.

Das Forschungsprojekt wird nicht finanziell unterstützt.

Il progetto di ricerca non è finanziato.

Le projet de recherche ne bénéficie d'aucun soutien financier.

The research project is not financially supported.

**Vertraulichkeit und Risiken**: Die Teilnahme ist freiwillig und kann jederzeit abgebrochen werden. Die Daten werden anonym erhoben und unterliegen strengen Datenschutzvorschriften. Somit bestehen für Sie keine Risiken.

**Riservatezza e rischi**: La partecipazione è volontaria e può essere annullata in qualsiasi momento. I dati vengono raccolti in forma anonima e sono soggetti a severe norme di protezione dei dati. Non ci sono quindi rischi per l'utente.

**Confidentialité et risques**: La participation est volontaire et peut être interrompue à tout moment. Les données sont collectées de manière anonyme et sont soumises à des règles strictes de protection des données. Il n'y a donc aucun risque pour vous.

**Confidentiality and risks**: Participation is voluntary and can be terminated at any time. The data is collected anonymously and is subject to strict data protection regulations. There are therefore no risks for you.

Mit der Teilnahme bezeugen Sie, dass Sie freiwillig teilnehmen und dass Sie die aufgeführten Informationen zur Kenntnis genommen haben.

Partecipando, confermate di partecipare volontariamente e di aver preso atto delle informazioni fornite.

En participant, vous attestez que vous êtes volontaire et que vous avez pris connaissance des informations mentionnées.

By participating, you confirm that you are participating voluntarily and that you have taken note of the information provided.

1. **Meldepraxis
   Pratica di reporting
   Pratique de déclaration
   Reporting practice**

**Q1** Haben Sie jemals bei einem Ihrer Patient:innen eine schwerwiegende oder bislang unbekannte unerwünschte Arzneimittelwirkung (UAW) vermutet oder diagnostiziert?

Ha mai sospettato o diagnosticato una reazione avversa al farmaco (ADR) grave o precedentemente sconosciuta in uno dei suoi pazienti?

Avez-vous déjà suspecté ou diagnostiqué un effet indésirable (EI) grave ou inconnu chez l'un de vos patients?
Have you ever suspected or diagnosed a serious or previously unknown adverse drug reaction (ADR) in one of your patients?

- Ja
  Sì
  Oui
  Yes
- Nein
  No
  Non
  No
- Keine Angabe
  Non specificato
  Aucune indication
  No specification

**Q2** Haben Sie schon einmal eine unerwünschte Arzneimittelwirkung gemeldet?

Ha mai segnalato una reazione avversa al farmaco?

Avez-vous déjà signalé un effet indésirable d'un médicament?
Have you ever reported an adverse drug reaction?

- Noch nie
  Mai prima d'ora
  Jamais encore
  Never before
- 1-3-mal
  1-3 volte
  1 à 3 fois
  1-3 times
- 4-10-mal
  4-10 volte
  4 à 10 fois
  4-10 times
- Mehr als 10-mal
  Più di 10 volte
  Plus de 10 fois
  More than 10 times
- Keine Angabe
  Non specificato
  Aucune indication
  No specification

**Q3** An welche Institution(en) haben Sie gemeldet?
A quale/i istituzione/i ha fatto riferimento?
À quelle(s) institution(s) avez-vous fait votre déclaration?
Which institution(s) have you reported to?

- Swissmedic
  Swissmedic
  Swissmedic
  Swissmedic
- Pharmazeutisches Unternehmen
  Azienda farmaceutica
  Société pharmaceutique
  Pharmaceutical company
- Regionales Zentrum (GE, VD, TI, BS, ZH, BE)
  Centro regionale (GE, VD, TI, BS, ZH, BE)
  Centre régional (GE, VD, TI, BS, ZH, BE)
  Regional center (GE, VD, TI, BS, ZH, BE)
- Kantonsarzt/Kantonsärztin
  Medico cantonale
  Médecin cantonal
  Cantonal doctor
- Kantonsapotheker/Kantonsapothekerin
  Farmacista cantonale
  Pharmacien cantonal/pharmacienne cantonale
  Cantonal pharmacist
- Sonstige [Bitte angeben:]
  Altro [Si prega di specificare:]
  Autre [Veuillez préciser:]
  Other [Please specify:]

1. **Wissen über Meldesystem
   Conoscenza del sistema di reporting
   Connaissance du système de notification
   Knowledge of the reporting system**

**Q4** Über welche der folgenden Aspekte haben Sie im Rahmen Ihrer Berufsausbildung Informationen erhalten?
Su quali dei seguenti aspetti ha ricevuto informazioni nell'ambito della sua formazione professionale?
Parmi les aspects suivants, sur lesquels avez-vous reçu des informations dans le cadre de votre formation professionnelle?
Which of the following aspects did you receive information about as part of your vocational training?

- Die gesetzliche UAW-Meldepflicht für medizinischen Fachpersonen
  L'obbligo di segnalazione delle ADR per i professionisti della sanità
  L'obligation légale de déclaration des EI pour les professionnels de la santé
  The statutory ADR reporting obligation for healthcare professionals
- Institutionen, welche am Spontanmeldesystem beteiligt sind
  Istituzioni coinvolte nel sistema di segnalazione spontanea
  Institutions impliquées dans le système d'annonce spontanée
  Institutions involved in the spontaneous reporting system
- Den Ablauf einer UAW-Meldung
  La procedura di segnalazione delle ADR
  La procédure de déclaration d'un EI
  The procedure for reporting ADR
- Informationen darüber, welche Arzneimittelwirkungen gemeldet werden sollen
  Informazioni su quali reazioni avverse ai farmaci devono essere segnalate
  Des informations sur les effets des médicaments qui doivent être notifiés
  Information on which adverse drug reactions should be reported
- Hinweis, wo ich Fachinformationen abrufen kann (z.B. swissmedicinfo.ch)
  Informazioni su dove posso accedere a informazioni specializzate (ad es. swissmedicinfo.ch)
  Indication de l'endroit où je peux consulter des informations spécialisées (p. ex. swissmedicinfo.ch)
  Information on where I can access specialist information (e.g. swissmedicinfo.ch)

*(Antwortmöglichkeiten):*
Ja / Nein / Weiss nicht / Keine Angabe
Sì / No / Non lo so / Non specificato
Oui / Non / Ne sais pas / Aucune indication
Yes / No / Don't know / No specification

1. **Einstellung zum Meldesystem**

**Atteggiamento nei confronti del sistema di reporting
Attitude envers le système de déclaration
Attitude towards the reporting system**

**Q5** Welche Gründe könnten Sie davon abhalten, eine unerwünschte Arzneimittelwirkung zu melden?

Quali motivi potrebbero impedirvi di segnalare una reazione avversa al farmaco?

Quelles sont les raisons qui pourraient vous empêcher de déclarer un effet indésirable d'un médicament?
What reasons might prevent you from reporting an adverse drug reaction?

- Der Zeitaufwand ist mir zu hoch.
  Il tempo necessario è troppo per me.
  L'investissement en temps est trop important pour moi.
  The time involved is too much for me.
- Ich kenne das Spontanmeldesystem für unerwünschte Arzneimittelwirkungen nicht.
  Non conosco il sistema di segnalazione spontanea delle reazioni avverse ai farmaci.
  Je ne connais pas le système d'annonce spontanée des effets indésirables des médicaments.
  I am not familiar with the spontaneous reporting system for adverse drug reactions.
- Ich kenne die Institutionen, welche am Spontanmeldesystem beteiligt sind, nicht.
  Non conosco le istituzioni coinvolte nel sistema di segnalazione spontanea.
  Je ne connais pas les institutions qui participent au système d'annonce spontanée.
  I do not know the institutions involved in the spontaneous reporting system.
- Ich halte das Melden von UAW nicht für sinnvoll.
  Non credo che abbia senso segnalare gli ADR.
  Je ne pense pas que la déclaration des EI soit utile.
  I don't think it makes sense to report ADRs.
- Mir fehlen die Anreize (z.B. ECTS oder Vergütung).
  Mi mancano gli incentivi (ad esempio ECTS o retribuzione).
  Il me manque des incitations (par ex. ECTS ou rémunération).
  I miss the incentives (e.g. ECTS or remuneration).
- Ich bin mir unsicher, welche Arzneimittelwirkungen ich melden soll.
  Non sono sicuro degli effetti dei farmaci che dovrei segnalare.
  Je ne sais pas quels effets médicamenteux je dois signaler.
  I am unsure which drug effects I should report.
- Unklarheit über die Bestimmungen zum Datenschutz der Patient:innen
  Mancanza di chiarezza sulle disposizioni relative alla protezione dei dati dei pazienti
  Manque de clarté sur les dispositions relatives à la protection des données des patients
  Lack of clarity about the provisions on patient data protection
- Unklarheit über meinen eigenen Datenschutz
  Non sono sicuro della mia protezione dei dati
  Manque de clarté sur ma propre protection des données
  Uncertainty about my own data protection
- Ich bin mir unsicher, ob eine Meldung juristische Konsequenzen haben kann.
  Non sono sicuro che una segnalazione possa avere conseguenze legali.
  Je ne suis pas sûr qu'un rapport puisse avoir des conséquences juridiques.
  I am unsure whether a report can have legal consequences.

*(Antwortmöglichkeiten):*
Stimme nicht zu / Neutral / Stimme zu / Keine Angabe

Non sono d'accordo / Neutrale / Sono d'accordo / Non specificato

Pas d'accord / Neutre / D'accord / Aucune indication

Disagree / Neutral / Agree / No specification

**Q6** Wie viele Minuten wären Sie maximal bereit, für das Melden einer unerwünschten Arzneimittelwirkung aufzubringen? 
[Bitte geben Sie Ihre Antwort in Anzahl Minuten ein.]

Qual è il numero massimo di minuti che sarebbe disposto a dedicare alla segnalazione di una reazione avversa a un farmaco? 
[Indicare la risposta in numero di minuti].

Quel est le nombre maximal de minutes que vous seriez prêt à consacrer à l'annonce d'un effet indésirable d'un médicament? 
[Veuillez indiquer votre réponse en nombre de minutes].
What is the maximum number of minutes you would be willing to spend on reporting an adverse drug reaction?

[Please enter your answer in number of minutes.]

**Q7** Über welchen Weg würden Sie eine unerwünschte Arzneimittelwirkung vorzugsweise melden?
Come preferisce segnalare una reazione avversa al farmaco?

Par quel moyen préfèreriez-vous annoncer un effet indésirable d'un médicament?

How would you prefer to report an adverse drug reaction?

- Online-Meldebogen über Website
  Modulo di registrazione online tramite sito web
  Formulaire d'inscription en ligne via le site web
  Online registration form via website
- Über ein elektronisches Portal
  Tramite un portale elettronico
  Via un portail électronique
  Via an electronic portal
- Per E-Mail
  Per e-mail
  Par e-mail
  By e-mail
- Telefonisch
  Per telefono
  Par téléphone
  By telephone
- Über eine App
  Tramite un'app
  Via une application
  Via an app
- Praxissoftware/Apothekensoftware
  Software per studi medici/ farmacie
  Logiciels pour cabinets médicaux/pharmacies
  Practice software/pharmacy software
- Meldebogen per Fax
  Modulo di registrazione via fax
  Formulaire de déclaration par fax
  Registration form by fax
- Meldebogen per Post
  Modulo di registrazione per posta
  Formulaire d'inscription par courrier
  Registration form by post
- Sonstiges [Bitte angeben:]
  Altro [Si prega di specificare:]
  Autre [Veuillez préciser:]
  Other [Please specify:]

**Q8** Welche Form der Rückantwort wäre für Sie hilfreich?
Quale forma di risposta sarebbe utile per voi?

Quelle forme de réponse vous serait utile?

What form of response would be helpful for you?

- Eingangsbestätigung
  Conferma di ricezione
  Accusé de réception
  Confirmation of receipt
- Informationen zum verdächtigen Arzneimittel (z.B. Fachinformation)
  Informazioni sul medicinale sospetto (ad es. riassunto delle caratteristiche del prodotto)
  Informations sur le médicament suspect (par ex. information professionnelle)
  Information on the suspected medicinal product (e.g. Summary of Product Characteristics)
- Empfehlungen zur weiteren Therapie
  Raccomandazioni per un'ulteriore terapia
  Recommandations pour la suite de la thérapie
  Recommendations for further therapy
- Bewertung des Schweregrades / Kausalzusammenhangs
  Valutazione del grado di gravità / rapporto di causalità
  Évaluation de la gravité / du lien de causalité
  Assessment of the degree of severity / causal relationship
- Informationen zur Arzneimittelwirkung (z.B. Datenbankrecherche, wissenschaftliche Publikation)
  Informazioni sugli effetti dei farmaci (ad es. ricerca su database, pubblicazione scientifica)
  Informations sur les effets des médicaments (par ex. recherche dans une base de données, publication scientifique)
  Information on drug effects (e.g. database research, scientific publication)
- Informationen darüber, was mit den gemeldeten Daten passiert (Bearbeitungsstatus)
  Informazioni su cosa succede ai dati segnalati (stato di elaborazione)
  Informations sur ce qui se passe avec les données déclarées (statut de traitement)
  Information on what happens to the reported data (processing status)
- Informationen darüber, wie oft diese unerwünschte Arzneimittelwirkung bereits gemeldet wurde
  Informazioni sulla frequenza con cui questa reazione avversa al farmaco è già stata segnalata
  Des informations sur le nombre de fois où cet effet indésirable a déjà été signalé
  Information on how often this adverse drug reaction has already been reported
- Ich benötige keine Rückantwort.
  Non ho bisogno di una risposta.
  Je n'ai pas besoin de réponse.
  I do not need a reply.
- Sonstiges [Bitte angeben:]
  Altro [Si prega di specificare:]
  Autre [Veuillez préciser:]
  Other [Please specify:]

**Q9** Gelegentlich ergeben sich bei der Bearbeitung von UAW-Meldungen Rückfragen. 
Welche der folgenden Aussagen treffen für Sie zu?
Occasionalmente, durante l'elaborazione dei rapporti ADR, sorgono delle domande.
Quali delle seguenti affermazioni si applicano a voi?
Il arrive que des questions soient posées lors du traitement des déclarations d'EI.
Laquelle des affirmations suivantes s'applique à votre cas?
Occasionally, queries arise during the processing of ADR reports.

Which of the following statements apply to you?

- Ich würde auf Nachfrage weitere Unterlagen zur Verfügung stellen (z.B. Entlassungsberichte, Laborbefunde).
  Su richiesta, fornirò ulteriori documenti (ad es. relazioni di dimissione, risultati di laboratorio).
  Je fournirais d'autres documents sur demande (par ex. rapports de sortie, résultats de laboratoire).
  I would provide further documents on request (e.g. discharge reports, laboratory results).
- Ich wäre bereit, konkrete Fragen schriftlich zu beantworten.
  Sarei pronto a rispondere per iscritto a domande specifiche.
  Je serais prêt à répondre par écrit à des questions concrètes.
  I would be prepared to answer specific questions in writing.
- Ich finde die Nachfrage nach weiteren Zusatzinformationen lästig.
  Trovo fastidiosa la richiesta di informazioni aggiuntive.
  Je trouve que la demande d'informations complémentaires supplémentaires est agaçante.
  I find the demand for additional information annoying.
- Rückfragen werden von mir in der Regel nicht beantwortet.
  Di norma, non rispondo alle domande.
  En règle générale, je ne réponds pas aux demandes de précisions.
  I do not usually answer queries.

**Q10** Würden Sie eine unerwünschte Arzneimittelwirkung auch melden, wenn sie durch einen Medikationsfehler verursacht wurde?

Riferirebbe anche una reazione avversa a un farmaco se fosse causata da un errore di somministrazione?
Déclareriez-vous également un effet indésirable d'un médicament s'il était dû à une erreur de médication?
Would you also report an adverse drug reaction if it was caused by a medication error?

- Ja
  Sì
  Oui
  Yes
- Nein
  No
  Non
  No
- Nur anonym
  Solo anonimo
  Anonyme uniquement
  Anonymous only
- Keine Angabe
  Non specificato
  Aucune indication

No specification

1. **Informationsbedürfnis
   Necessità di informazioni
   Besoin d'information
   Need for information**

**Q11** Über welche Aspekte des bestehenden Spontanmeldesystems wünschen Sie sich weitere Informationen?
Su quali aspetti dell'attuale sistema di segnalazione spontanea desidera maggiori informazioni?

Sur quels aspects du système de notification spontanée existant souhaiteriez-vous obtenir davantage d'informations?
What aspects of the existing spontaneous reporting system would you like more information on?

- Den Sinn und Zweck der Meldung von unerwünschten Arzneimittelwirkungen.
  Lo scopo della segnalazione delle reazioni avverse ai farmaci.
  Le sens et l'objectif de la notification des effets indésirables des médicaments.
  The purpose of reporting adverse drug reactions.
- Den Effekt solcher Meldungen.
  L'effetto di tali messaggi.
  L'effet de tels messages.
  The effect of such messages.
- Die bestehenden Möglichkeiten, unerwünschte Arzneimittelwirkungen zu melden.
  Le opzioni esistenti per la segnalazione delle reazioni avverse ai farmaci.
  Les possibilités existantes de déclarer les effets indésirables des médicaments.
  The existing options for reporting adverse drug reactions.
- Den Ablauf der Meldung von unerwünschten Arzneimittelwirkungen.
  La procedura di segnalazione delle reazioni avverse ai farmaci.
  La procédure d'annonce des effets indésirables d'un médicament.
  The procedure for reporting adverse drug reactions.
- Informationen darüber, welche Arzneimittelwirkungen gemeldet werden sollen.
  Informazioni sugli effetti dei farmaci da segnalare.
  Des informations sur les effets des médicaments qui doivent être notifiés.
  Information on which drug effects should be reported.
- Informationen zur Relevanz von unerwünschten Arzneimittelwirkungen spezifisch für mein Fachgebiet.
  Informazioni sulla rilevanza delle reazioni avverse ai farmaci specifiche per la mia specialità.
  Informations sur la pertinence des effets indésirables des médicaments spécifiquement pour ma spécialité.
  Information on the relevance of adverse drug reactions specific to my specialty.
- Die juristischen Grundlagen der Meldung unerwünschter Arzneimittelwirkungen.
  La base legale per la segnalazione delle reazioni avverse ai farmaci.
  Les bases juridiques de l'annonce des effets indésirables des médicaments.
  The legal basis for reporting adverse drug reactions.
- Die gesetzliche Meldepflicht von medizinischen Fachpersonen.
  L'obbligo legale di segnalazione da parte degli operatori sanitari.
  L'obligation légale de déclaration des professionnels de la santé.
  The legal obligation of healthcare professionals to report.
- Datenschutzaspekte
  Aspetti di protezione dei dati
  Aspects relatifs à la protection des données
  Data protection aspects
- Sonstige [Bitte angeben:]
  Altro [Si prega di specificare:]
  Autre [Veuillez préciser:]
  Other [Please specify:]
- Ich benötige keine weiteren Informationen.
  Non ho bisogno di altre informazioni.
  Je n'ai pas besoin d'autres informations.
  I do not need any further information.

**Q12** Über welche Kanäle würden Sie sich weitere Informationen zum Spontanmeldesystem und dessen Sinn und Zweck wünschen?

Attraverso quali canali vorrebbe ricevere maggiori informazioni sul sistema di segnalazione spontanea e sulle sue finalità?

Par quels canaux souhaiteriez-vous recevoir plus d'informations sur le système d'annonce spontanée et sur sa raison d'être?
Through which channels would you like to receive more information about the spontaneous reporting system and its purpose?

- Monatliche Ärztezeitschrift
  Rivista medica mensile
  Revue médicale mensuelle
  Monthly medical journal
- pharmaJournal
  pharmaJournal
  pharmaJournal
  pharmaJournal
- Fortbildung
  Formazione continua
  Formation continue
  Further training
- E-Mail-Newsletter
  Newsletter via e-mail
  Bulletin d'information par e-mail
  E-mail newsletter
- Sonstige [Bitte angeben:]
  Altro [Si prega di specificare:]
  Autre [Veuillez préciser:]
  Other [Please specify:]
- Ich wünsche mir keine weiteren Informationen.
  Non desidero ricevere ulteriori informazioni.
  Je ne souhaite pas recevoir d'autres informations.
  I do not wish to receive any further information.
- Keine Angabe
  Non specificato
  Aucune indication
  Not specified

**Q13** Würden Sie einen Aus-, Weiterbildungs- bzw. Auffrischungskurs für Pharmakovigilanz in Anspruch nehmen?

Vorrebbe partecipare a un corso di formazione, perfezionamento o aggiornamento sulla farmacovigilanza?
Auriez-vous recours à un cours de formation, de perfectionnement ou de remise à niveau en matière de pharmacovigilance?
Would you take advantage of a training, further training or refresher course in pharmacovigilance?

- Nein
  No
  Non
  No
- Eher nein
  Piuttosto no
  Plutôt non
  Rather no
- Neutral (weder noch)
  Neutrale (nessuno dei due)
  Neutre (ni l'un ni l'autre)
  Neutral (neither)
- Eher ja
  Piuttosto sì
  Plutôt oui
  Rather yes
- Ja
  Sì
  Oui
  Yes
- Keine Angabe
  Non specificato
  Aucune indication
  Not specified

1. **Soziodemografie
   Dati socio-demografici
   Sociodémographie
   Sociodemographics**

⚠️ *(Disclaimer)*
Ihre demografischen Informationen werden nur zu statistischen Zwecken erfasst und haben keinen Einfluss auf Ihre persönliche Identifizierung oder Privatsphäre.

Le informazioni demografiche vengono raccolte solo a fini statistici e non hanno alcuna attinenza con l'identificazione personale o la privacy dell'utente.

Vos informations démographiques ne sont collectées qu'à des fins statistiques et n'ont aucune incidence sur votre identification personnelle ou votre vie privée.
Your demographic information is collected for statistical purposes only and has no bearing on your personal identification or privacy.

**Q14** In welchem Altersbereich befinden Sie sich?
In quale fascia d'età si trova?
Dans quelle tranche d'âge vous situez-vous?
What age range are you in?

- Unter 20 Jahre
  Meno di 20 anni
  Moins de 20 ans
  Under 20 years
- 20-29 Jahre
  20-29 anni
  20-29 ans
  20-29 years
- 30-39 Jahre
  30-39 anni
  30-39 ans
  30-39 years
- 40-49 Jahre
  40-49 anni

40-49 ans
40-49 years

- 50-59 Jahre
  50-59 anni

50-59 ans
50-59 years

- 60-69 Jahre
  60-69 anni
  60-69 ans
  60-69 years
- 70 Jahre oder älter
  70 anni o più
  70 ans ou plus
  70 years or older
- Keine Angabe
  Non specificato
  Aucune indication
  Not specified

**Q15** Welchem Geschlecht fühlen Sie sich zugehörig?
A quale genere sente di appartenere?

De quel sexe vous sentez-vous appartenir?
Which gender do you feel you belong to?

- Männlich
  Uomo
  Mâle
  Male
- Weiblich
  Donna
  Femme
  Female
- Nichtbinär/drittes Geschlecht
  Non binario/terzo genere
  Non-binaire/troisième sexe
  Non-binary/third gender
- Keine Angabe
  Non specificato
  Aucune indication
  Not specified

**Q16** Welcher Berufsgruppe gehören Sie an?
A quale gruppo professionale appartenete?
Quelle est votre profession?
Which professional group do you belong to?

- Hausarzt/Hausärztin
  Medico di famiglia
  Médecin de famille
  General practitioner
- Niedergelassene:r Spezialist:in
  Specialista affermato
  Spécialiste établi(e)
  Specialist in private practice
- Arzt/Ärztin im Spital
  Medico in ospedale
  Médecin à l'hôpital
  Doctor in a hospital
- Arzt/Ärztin in der Forschung
  Dottore di ricerca
  Médecin dans la recherche
  Doctor in research
- Apotheker/Apothekerin in einer öffentlichen Apotheke
  Farmacista in una farmacia pubblica
  Pharmacien(ne) dans une pharmacie publique
  Pharmacist in a public pharmacy
- Apotheker/Apothekerin in einer Spitalapotheke
  Farmacista in una farmacia ospedaliera
  Pharmacien(ne) dans une pharmacie d'hôpital
  Pharmacist in a hospital pharmacy
- Apotheker/Apothekerin in der Forschung
  Farmacista nella ricerca
  Pharmacien(ne) chercheur(euse)
  Pharmacist in research
- Sonstiges [Bitte angeben:]
  Altro [Si prega di specificare:]
  Autre [Veuillez préciser:]
  Other [Please specify:]
- Keine Angabe
  Non specificato
  Aucune indication
  No specification

**Q16b** In welchem Fachbereich arbeiten Sie hauptsächlich?

In quale area specialistica lavora principalmente?
Dans quel domaine travaillez-vous principalement?
In which specialist area do you mainly work?

- Allgemeine Innere Medizin
  Medicina interna generale
  Médecine interne générale
  General internal medicine
- Anästhesiologie
  Anestesiologia
  Anesthésiologie
  Anesthesiology
- Chirurgie
  Chirurgia
  Chirurgie
  Surgery
- Gynäkologie und Geburtshilfe
  Ginecologia e ostetricia
  Gynécologie et obstétrique
  Gynecology and obstetrics
- Kinder- und Jugendmedizin
  Pediatria e medicina degli adolescenti
  Pédiatrie et médecine de l'adolescence
  Pediatrics and adolescent medicine
- Ophthalmologie
  Oftalmologia
  Ophtalmologie
  Ophthalmology
- Orthopädische Chirurgie
  Chirurgia ortopedica
  Chirurgie orthopédique
  Orthopaedic surgery
- Praktischer Arzt/Ärztin
  Medico di base
  Médecin praticien
  Practical doctor
- Psychiatrie und Psychotherapie
  Psichiatria e psicoterapia
  Psychiatrie et psychothérapie
  Psychiatry and psychotherapy
- Radiologie
  Radiologia
  Radiologie
  Radiology
- Ohne Hauptfachgebiet
  Senza specializzazione principale
  Sans spécialité principale
  Without main subject area
- Sonstiges [Bitte angeben:]
  Altro [Si prega di specificare:]
  Autre [Veuillez préciser:]
  Other [Please specify:]
- Keine Angabe
  Non specificato
  Aucune indication
  Not specified

**Q16b** Welche der folgenden Weiterbildungen haben Sie abgeschlossen?
Quali dei seguenti programmi di formazione ha completato?

Parmi les formations suivantes, lesquelles avez-vous suivies?
Which of the following training courses have you completed?

- Fachapotheker:in Spitalpharmazie FPH
  Farmacista specialista in farmacia ospedaliera FPH
  Pharmacien(ne) spécialisé(e) en pharmacie hospitalière FPH
  Specialist pharmacist in hospital pharmacy FPH
- Fachapotheker:in Offizinpharmazie FPH
  Farmacista specialista in farmacia FPH
  Pharmacien(ne) spécialisé(e) en pharmacie d'officine FPH
  Specialist pharmacist in pharmacy FPH
- FPH Klinische Pharmazie
  Farmacia clinica FPH
  FPH Pharmacie clinique
  FPH Clinical Pharmacy
- CAS Klinische Pharmazie
  Farmacia clinica CAS
  CAS Pharmacie clinique
  CAS Clinical Pharmacy
- DAS Spitalpharmazie
  Farmacia ospedaliera DAS
  DAS Pharmacie hospitalière
  DAS Hospital Pharmacy
- FPH Zertifikat Pharmazeutische Betreuung von Institutionen im Gesundheitswesen
  Certificato FPH in assistenza farmaceutica per le istituzioni sanitarie
  Certificat FPH Encadrement pharmaceutique d'institutions du secteur de la santé
  FPH certificate Pharmaceutical support for healthcare institutions
- Andere [Bitte angeben:]
  Altro [Si prega di specificare:]
  Autre [Préciser:]
  Other [Please specify:]
- Ich befinde mich zurzeit in der Weiterbildung.
  Attualmente sono in fase di perfezionamento.
  Je suis actuellement en formation continue.
  I am currently undergoing further training.
- Ich habe keine der oben genannten Weiterbildungen abgeschlossen.
  Non ho completato nessuno dei programmi di formazione sopra menzionati.
  Je n'ai terminé aucune des formations continues mentionnées ci-dessus.
  I have not completed any of the training courses mentioned above.
- Keine Angabe
  Non specificato
  Aucune indication
  No specification

**Q17** Seit wie vielen Jahren sind Sie bereits nach dem Studium in Ihrem Beruf tätig? 
[Bitte geben Sie Ihre Antwort in Anzahl Jahren an]
Da quanti anni lavora nella sua professione dopo la laurea? 
[Indicare la risposta in numero di anni].
Depuis combien d'années exercez-vous votre profession après vos études? 
[Veuillez indiquer votre réponse en nombre d'années]
How many years have you been working in your profession after graduating?

1. **Verbesserungen
   Miglioramenti
   Améliorations
   Improvements**

**Q18** Haben Sie Vorschläge/Anregungen zur Verbesserung des aktuellen Meldesystems?

Avete suggerimenti per migliorare l'attuale sistema di reporting?
Avez-vous des propositions/suggestions pour améliorer le système de signalement actuel?
Do you have any proposals/suggestions for improving the current reporting system?

**Q19** Haben Sie sonstige Anmerkungen/Kommentare?

Avete altre osservazioni/commenti?
Avez-vous d'autres remarques/commentaires?
Do you have any other remarks/comments?

***Schluss***

Vielen Dank! Sie sind am Ende der Befragung angelangt. 
Ihre Rückmeldungen wurden erfasst und unterstützen das Forschungsprojekt, wofür wir Ihnen sehr dankbar sind.

Grazie mille! Avete raggiunto la fine del sondaggio. 
Il vostro feedback è stato registrato e supporta il progetto di ricerca, per cui vi siamo molto grati.

Merci beaucoup! Vous êtes arrivés à la fin de l'enquête.

Vos réactions ont été enregistrées et soutiennent le projet de recherche, ce dont nous vous sommes très reconnaissants.

Thank you very much! You have reached the end of the survey.

Your feedback has been recorded and supports the research project, for which we are very grateful.
